# Exploring the Relationships between Macrofungi Diversity and Major Environmental Factors in Wunvfeng National Forest Park in Northeast China

**DOI:** 10.3390/jof8020098

**Published:** 2022-01-20

**Authors:** Yonglan Tuo, Na Rong, Jiajun Hu, Guiping Zhao, Yang Wang, Zhenhao Zhang, Zhenxiang Qi, Yu Li, Bo Zhang

**Affiliations:** Engineering Research Center of Edible and Medicinal Fungi, Ministry of Education, Jilin Agricultural University, Changchun 130118, China; tuoyonglan66@163.com (Y.T.); immortalRong@163.com (N.R.); waynehu1993@126.com (J.H.); guipingz6@163.com (G.Z.); lesireyang@163.com (Y.W.); zzhzz34@163.com (Z.Z.); qzx7007@163.com (Z.Q.)

**Keywords:** community composition, conservation, edaphic variables, forest type, macrofungal species richness

## Abstract

In this paper, we analyze the macrofungi communities of five forest types in Wunvfeng National Forest Park (Jilin, China) by collecting fruiting bodies from 2019–2021. Each forest type had three repeats and covered the main habitats of macrofungi. In addition, we evaluate selected environmental variables and macrofungi communities to relate species composition to potential environmental factors. We collected 1235 specimens belonging to 283 species, 116 genera, and 62 families. We found that *Amanitaceae*, *Boletaceae*, *Russulaceae*, and *Tricholomataceae* were the most diverse family; further, *Amanita*, *Cortinarius*, *Lactarius*, *Russula*, and *Tricholoma* were the dominant genera in the area. The macrofungi diversity showed increasing trends from *Pinus koraiensis* Siebold et Zuccarini forests to *Quercus mongolica* Fischer ex Ledebour forests. The cumulative species richness was as follows: *Q. mongolica* forest A > broadleaf mixed forest B > *Q. mongolica*, *P. koraiensis* mix forest D (*Q. mongolica* was the dominant species) > *Q. mongolica* and *P. koraiensis* mix forest C (*P. koraiensis* was the dominant species) > *P. koraiensis* forest (E). Ectomycorrhizal fungi were the dominant functional group; they were mainly in forest type A and were influenced by soil moisture content and *Q. mongolica* content (*p* < 0.05). The wood-rotting fungus showed richer species diversity than other forest types in broadleaf forests A and B. Overall, we concluded that most fungal communities preferred forest types with a relatively high *Q. mongolica* content. Therefore, the deliberate protection of *Q. mongolica* forests proves to be a better strategy for maintaining fungal diversity in Wunvfeng National Forest Park.

## 1. Introduction

Fungal communities are essential for forest ecosystems and have many functions [1,2]. Ectomycorrhizal fungi (EM) participate in the soil nutrient cycle of forest ecosystems and promote the host plant’s absorption of nutrients, such as nitrogen, phosphorus, and water, thereby maintaining the above-ground primary productivity of the forest ecosystem [3]. Saprotrophic fungi can degrade wood components (i.e., lignin, cellulose, and hemicellulose [4]), and they are considered essential wood-decay-promoting organisms. These functions indicate a crucial role in maintaining the forest ecosystem’s stability [5].

Many biotic and abiotic factors can affect the diversity and composition of fungal communities [6,7]. The composition of EM is strongly influenced by the soil’s nitrogen content [8,9], pH [10], temperature and moisture [11,12], the species composition of the host trees [13,14], and by the seasons [15]. Fungal communities living on the wood are closely dependent upon environmental factors, such as the amount, diameter, and stage of wood decomposition [16], wood chemistry [17], age [18], and tree species [19]. Factors influencing terricolous saprotrophic communities include litter quantity and pH [20], soil P content [21,22], plant species [23,24], and temperature [25]. The processes of natural or human-induced change in the vegetation composition of forests are also important drivers of fungal diversity [26,27,28], as they are associated with significant changes in litter and soil quality in the long term [29,30,31].

Our study sites are all within the Wunvfeng National Forest Park in the southeastern mountainous region of the Jilin Province, China. The reserve was established in 1992 and is part of the Changbai Mountain system. The original vegetation included *Quercus mongolica* Fischer ex Ledebour forests and mixed broadleaf forests, which changed when *Pinus koraiensis* Siebold et Zuccarini was planted in the 1980s [32]. Currently, the vegetation composition includes a *P. koraiensis* forest, and *P. koraiensis*–*Q. mongolica* mixed forests provide a unique opportunity to investigate the fungal communities of different forest types under the same climatic conditions. Usually, *Pinus* species are dependent upon macrofungi in symbiotic associations, which are essential for their growth and survival [33] because symbiotic associations facilitate the trees’ uptake of water and nutrients [34,35]. Some specific fungal species may be found in relatively stable *Pinus* forests. Thus, understanding the distribution characteristics of fungal communities in planted and native forests can provide better strategies for fungal diversity conservation, especially for the deliberate conservation of forest types that host a more significant number of fungal species. In this context, we conducted a three-year survey of macrofungi in different vegetation types in Wunvfeng National Forest Park. We study the relationship between selected environmental factors and community composition. We aim to investigate whether fungal communities differ among the five forest types and whether native forests dominated by *Q. mongolica* are potentially associated with fungal communities.

## 2. Materials and Methods

### 2.1. Study Area Description

Our study site, Wunvfeng National Forest Park, covers an area of 6867 hm^2^ [36] and is situated in Ji’an City in the extreme southeast part of the Jilin Province, Northeast China (126°2′21″–126°17′57″ E, 41°11′37″–41°21′40″ N). The area is characterized by a temperate continental monsoon climate with a mean annual precipitation of 950 mm with peaks from June–August and a mean annual air temperature of 6.5 °C [36]. The forest cover is 95%, and the dominant tree species is *Q. mongolica*. *P. koraiensis* was planted as a non-native tree species in 1980. At present, it has formed stable forest communities. Dark-brown soil [37] is the most frequent soil type. The fundamental geomorphological units belong to the Changbai Mountains, ranging in altitude from 500–1500 m [38].

### 2.2. Fruiting Body Sampling

**Figure 1:**
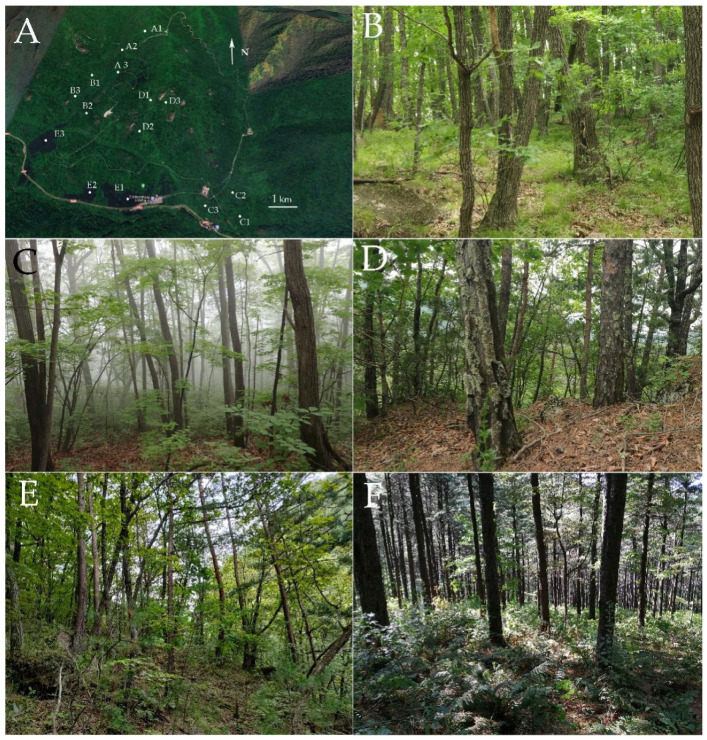
Sampling plot distribution and forest types in Wunvfeng National Forest Park. Note: Sampling plot distribution (**A**); Plots A1, A2, A3 = *Q. mongolica forest* (**B**); Plots B1, B2, B3 = Broad-leaved forest (**C**); Plots C1, C2, C3 = *P. koraiensis* and *Q. mongolica* mixed forest (*P. koraiensis* is the dominant species (**D**)); Plots D1, D2, D3 = *P. koraiensis* and *Q. mongolica* mixed forest, (*Q. mongolica* is the dominant species (**E**)); Plots E1, E2, E3 = *P. koraiensis* forest (**F**).

We designated five following forest types: A, B, C, D, and E. Three 50 m × 50 m sampling plots with at least 500 m distance between them were located within each forest type (pictures of each forest type are provided in Figure 1).

*Quercus mongolica* site (A): Old-growth *Q. mongolica* forests with the original habitat, and no fallen or standing deadwood has been removed. There are 275 *Q. mongolica* with a coverage rate of 96.15%.

Broad-leaved forest site (B): Mixed broadleaved forest (uneven-aged), with *Q. mongolica*, *Tilia amurensis* Rupr., *Fraxinus mandshurica* Rupr., *Acer palmatum* Thunb., *Juglans regia* L., close to nature managed. There are 158 *Q. mongolica* with a coverage rate of 48.46%.

*Pinus koraiensis* and *Quercus mongolica* mixed sites (C): 30–50-year-old *P. koraiensis* forest, close to nature managed, and naturally grown *Q. mongolica*. *P. koraiensis* was artificially grown and is the dominant species. There are 42 *Q. mongolica* with a coverage rate of 18.03%. 

*Quercus mongolica* and *Pinus koraiensis* mixed sites (D): 30–60-year-old *Q. mongolica* forests, close to nature managed, and artificially grown *P. koraiensis*. *Q. mongolica* is the dominant species. There are 183 *Q. mongolica* with a coverage rate of 74.69%.

*Pinus. koraiensis sites* (E): Single species; 40-year-old *P. koraiensis* forests planted in 1980, close to nature managed, no fallen trees removed. There are zero *Q. mongolica* with a coverage rate of 0%.

**Figure 2:**
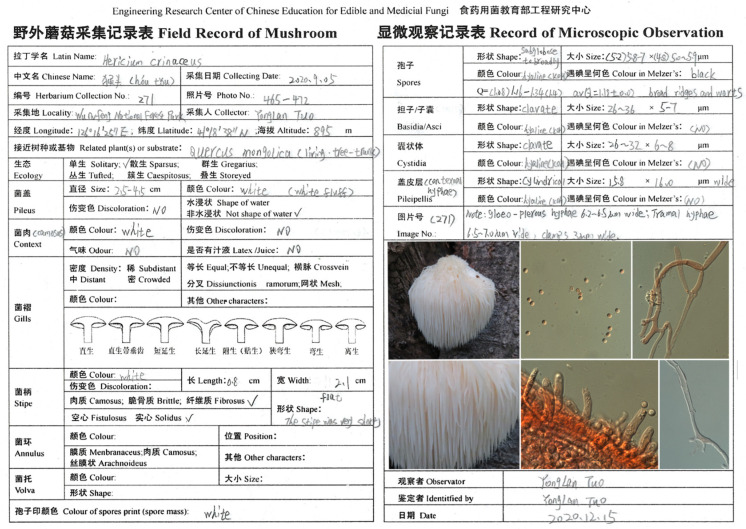
Field record of mushrooms and microscopic characteristic observation.

We collected samples 20–25 times per month from June to October 2019–2021. We randomly acquired macrofungi from each plot. We photographed the specimens in the field using a Canon EOS 800D digital camera and recorded fresh morphological characteristics and ecological characteristics (Figure 2). We selected the context or stipe tissue (1–2 g) of the same specimen when it was fresh and stored it in a sealed bag with silica gel for DNA extraction; we dried them in an oven (45–50 °C) and placed them in specimen boxes. We then took a morphotype of each specimen to the laboratory and used it for morphological species identification.

### 2.3. Soil Sampling, Analysis and Environmental Data Collection

We collected soil samples four times per month during July–September 2020. After cleaning and removing plant material and debris from the surface, we collected individual soil samples from the center and 4 corners in 15 plots using an auger (5 cm radius, 5 cm depth). We mixed the soil samples from the same plots well and placed them in sealed bags. After removing impurities, we enclosed the fresh samples (20 g) from each clod in an aluminum box. We dried the samples to a constant weight in an oven at 105 °C to measure water content (SWC); we used natural air-dried composite samples (200 g) for each plot to analyze for pH, organic matter (SOM), available phosphorus (P), effective nitrogen (N), and available potassium (K) using the method described by Xing et al. [39]. Finally, we averaged the data from three plots of the five fore for subsequent analysis. 

We obtained the temperature and relative humidity of the air and soil temperature from July–September 2020 from meteorological monitoring sites in the forest park. The tested results for the soil are included in Table 1.

### 2.4. Species Identification

We identified the macrofungi using morphological observations methods. We used molecular methods for the species that were morphologically difficult to identify. We measured different microscopic structures of taxonomic importance (e.g., spores, basidia, cystidia) [40]. We examined the morphological features of the fruiting bodies using appropriate monographs, including by Li et al. [41] and Liu et al. [42], to identify each macrofungi specimens. The specimens are currently housed in the Herbarium of Mycology of Jilin Agricultural University (HMJAU), Changchun, China.

Molecular identification involved sequencing the internal transcribed spacer (ITS). For this, we extracted the DNA of the macrofungi using a NuClean Plant Genomic DNA Kit (Cowin Biosciences, Taizhou, China), following the manufacturer’s instructions. We conducted final elutions in a total volume of 50 μL. We showed a polymerase chain reaction (PCR) with the primer pairs ITS-1F and ITS-4 [43]; finally, we sequenced the PCR products using the Sanger method. We conducted the PCR in 25 μL reactions consisting of 2 μL genomic DNA, 0.5 μL Taq, and one μL upstream and downstream primers, respectively. We used 14.5 μL ddH_2_O, five μL 5 × PCR buffer, and 1 µL dNTP in the PCR reactions that we ran under the following conditions: 95 °C for 3 min, followed by 35 cycles of 94 °C for 40 s, 55 °C for 45 s, 72 °C for 1.5 min, and a final extension step at 72 °C for 6 min before storage at 4 °C We purified the PCR products and sequenced them at Sangon Biotech Co., Ltd. (Shanghai, China). We performed molecular identification via BLAST comparisons. Species with >98% sequence similarity were also identified with morphological characteristics. GenBank accession numbers obtained are provided in Appendix B.

We identified ecological functions (ectomycorrhizal fungi; soil saprotroph; wood-decaying fungi; litter saprotroph; dung saprotroph; endophyte-insect pathogen) at the genera level using a FUNGuild (available online: http://www.funguild.org (accessed on 18 November 2021) search; these can be found in Appendix B. We classified macrofungi into eight types (agarics; large ascomycetes; boletes; polyporoid fungi; coral fungi; gasteoid fungi; jelly fungi; hydnaceous fungi, and cantharelloid fungi) according to the method of Li et al. [41]. The fungal nomenclature follows the Index Fungorum (available online: http://www.indexfungorum.org (accessed on 15 November 2021)). Setting scientific names at all taxonomic ranks in italics facilitates quick recognition in scientific papers [44].

### 2.5. Statistical Analysis

We used three alpha diversity indices to analyze the community composition of the macrofungi. The Menhinick richness index (R) reflected the species richness of the community. The Shannon index (D) reflected the diversity of the community species. Pielou’s evenness index (E) reflected the distribution of the number of individuals in each species. The diversity index formulae were as follows:

(1) $$R=S/\sqrt{N}$$

D = −∑*P_i_* ln (*P_i_*) (2)

E = H’/lnS; H’= −∑*P_i_* ln (*P_i_*) (3)

where *P_i_* is the proportion of species *i* to the total number of individuals of all species in the plot; ln is the natural logarithm; S is the total number of species in the plot; and N is the total number of individuals observed in the plot.

We analyzed the relationships between ectomycorrhizal fungi communities and selected variables using the canonical correlation analysis (CCA) from Canoco 5.0 [45]. We first used detrended correspondence analysis (DCA) to determine the appropriate model for direct gradient analysis. The results indicated that a unimodal model (gradient lengths > 3 standard units) would best fit our study data; we utilized CCA. Furthermore, we tested explanatory variables using the Monte Carlo permutation test provided by Canoco 5.0 software (with 999 randomizations). The species data matrix for the CCA analysis was based on the presence–absence data of ectomycorrhizal fungi species in each forest type (three-year accumulation of the five forest types).

We used Origin 9.0 software to construct species stacked histograms at the genera level [46] to compare community compositions of the macrofungi species in the five forests and provide the relative proportion of macrofungi species richness (data include the number of species at the genera level in each forest type). Additionally, we generated pie charts, Venn diagrams, and species accumulation curves using Hiplot (available online: https://hiplot.com.cn/basic/venn (accessed on 20 October 2021)). The pie chart data were derived from the number of macrofungi types. The Venn diagram data included the species in each forest type. The accumulation curve data consisted of the cumulative number of species per collection.

## 3. Results

### 3.1. Species Richness

We collected 1235 specimens from 5 forest types, 940 (76.11%) of which we identified at the species level, and we classified these into 283 fungal species. We identified 244 species based on morphology and 39 species using morphology and molecular methods (Appendix B). The unidentified sporocarps were not part of our further analysis. We classified the macrofungi species into 116 genera, 62 families, 18 orders, and 2 phyla. Basidiomycota was the dominant phylum, divided into 12 orders, 50 families, 102 genera, and 265 species. Ascomycota was divided into 6 orders, 12 families, 14 genera, and 18 species. The *Russulaceae* was the most diverse family with 36 different species, followed by *Tricholomataceae* (21 species), *Boletaceae* (19 species), and *Amanitaceae* (16 species). Together, these accounted for 32.51% of the total collected species. The most abundant genera were *Amanita, Cortinarius, Lactarius,* and *Russula*. The *Agaricales* were the most prevalent order in the five forest types (59.36%). In terms of the trophic groups, most of the species were ectomycorrhizal fungi (47%), followed by wood-decaying fungi (20.14%) and soil saprotrophs (18.37%).

### 3.2. Macrofungal Types 

**Figure 3:**
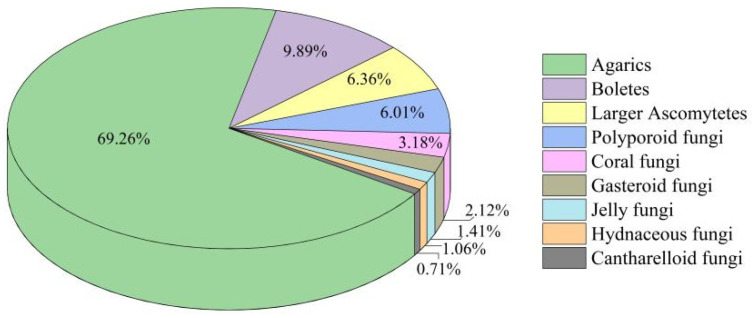
The proportion of different macrofungal types.

The most significant genera of *Agarics* accounted for 69.26% of the identified species, followed by boletes, larger ascomytetes, and polyporoid fungi, accounting for 9.89%, 6.36%, and 6.01% of the identified species, respectively. In contrast, hydnaceous and cantharelloid fungi were less abundant, accounting for 1.06% and 0.71%, respectively (see Figure 3). For more detailed information, see Appendix B. For images of some species, see Appendix A.

### 3.3. Analysis of Dominant Families and Genera

Among the identified species, there were 9 dominant families (number of species ≥10 species) of macrofungi (Table 2). The *Russulaceae* was the most diverse family. In addition, 53 families contained less than 10 species, accounting for 85.48% of the families and 48.06% of the identified species (Appendix B).

Among the identified species, there were 15 dominant genera (number of species ≥ 5 species) of macrofungi (Table 3). The *Amanita, Lactarius*, and *Russula* were the most diverse genera. In addition, 34 genera contained 2–4 species, accounting for 29.31% of the genera and 29.68% of the identified species; 67 of the genera contained only 1 species, accounting for 57.76% of the genera and 23.67% of the identified species (Appendix B).

### 3.4. Forest Type and Species Composition

**Figure 4:**
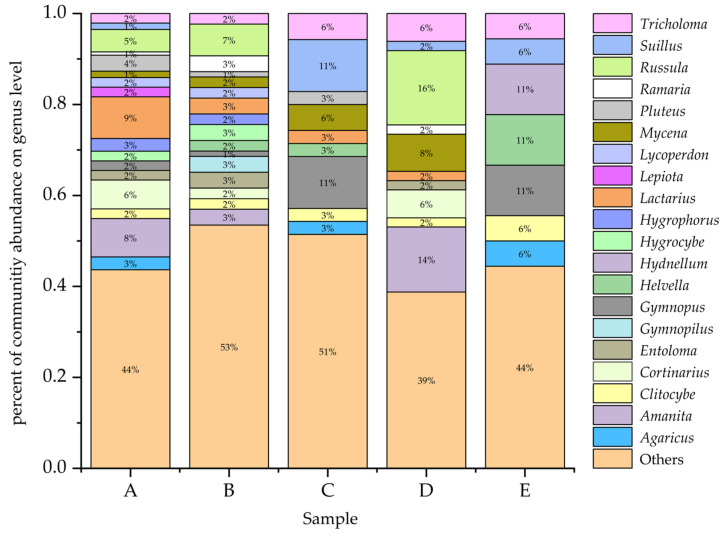
The relative proportions of macrofungi taxa at the genera level in five forest types. (**A**): *Q. mongolica* forest; (**B**): Broad-leaved forest; (**C**): *P. koraiensis* and *Q. mongolica* mixed forest with *P. koraiensis* being the dominant species; (**D**): *P. koraiensis* and *Q. mongolica* mixed forest with *Q. mongolica* being the dominant species; (**E**): *Pinus koraiensis* forest.

The community of macrofungi was different among the five forest types (Figure 4). The species richness increased from E (18 species, 6.36%) < C (35 species, 12.37%) < D (49 species, 17.31%) < B (86 species, 30.39%) < A (142 species, 50.18%). The *Lactarius* (13 species), *Amanita* (12 species), *Coprinellus* (10 species), and *Russula* (7 species) were the most species-rich genera in forest type A. The *Russula* (6 species) and *Gymnopilus* (4 species) were the most species-rich genera in forest type B. The *Gymnopus* (4 species) and *Suillus* (4 species) were the most species-rich genera in forest type C. The *Russula* (8 species), *Amanita* (7 species), and *Mycena* (4 species) were the most species-rich genera in forest type D. Finally, the *Gymnopus* (2 species), *Helvella* (2 species), and *Hydnellum* (2 species) were the relatively abundant genera in forest type E. More detailed information is shown in Appendix B.

### 3.5. Cumulative Abundance of Macrofungi in Five Forest Types

**Figure 5:**
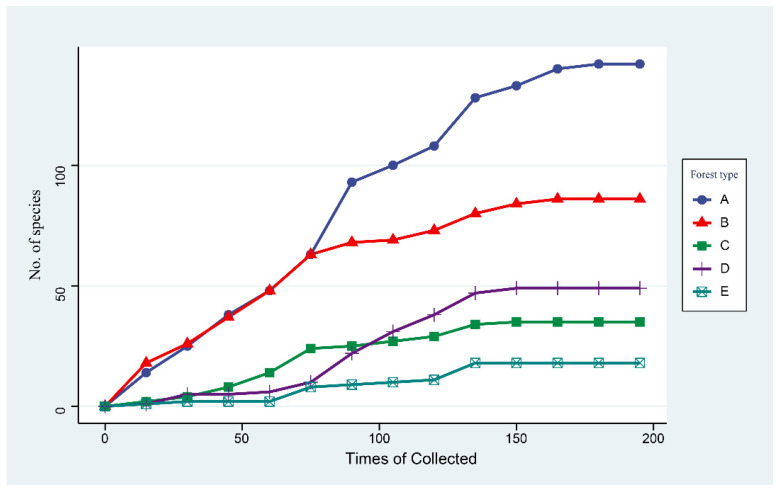
Sample-based rarefaction curves (n = 195 observations per forest type within 3 years). (**A**): *Q. mongolica* forest; (**B**): broad-leaved forest; (**C**): *P. koraiensis* and *Q. mongolica* mixed forest with *P. koraiensis* being the dominant species; (**D**): *P. koraiensis* and *Q. mongolica* mixed forest with *Q. mongolica* being the dominant species; (**E**): *P. koraiensis* forest.

The accumulation curves for the species identified in the five forests show a steady increase with more samplings (Figure 5). We reached saturation of macrofungi richness after 150 surveys. The species accumulation curves of A (*Q. mongolica* forest) and B (broad-leaved forest) showed relatively steep upward slopes and produced higher macrofungi abundance values than the other forests. Nevertheless, forest type A (*Q. mongolica* forest) obtained the highest macrofungi diversity values.

**Figure 6:**
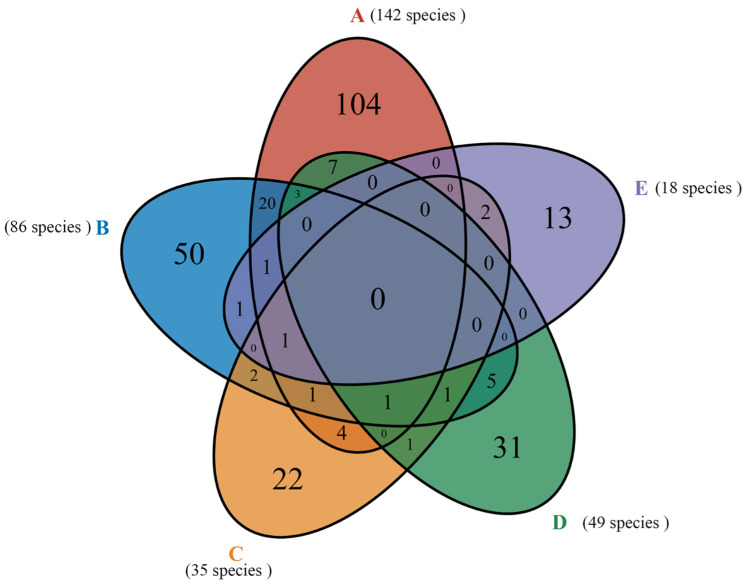
A Venn diagram of 283 fungal species shows shared and unique fungi for the five forest types. The numbers in parentheses are the values of all observed fungi in each forest type studied (cumulative species richness).

Two genera (*Clitocybe* and *Tricholoma*) were shared in five forest types, but no species were shared. The unique species (found only in 1 forest) increased from E < C < D < B < A and consisted of 13, 22, 31, 50, and 104 fungal species, respectively (Figure 6). Forest type A shared 27 species with B, 7 species with C, 11 species with D, and 2 species with E. Forest type B shared six species with C, eleven species with D, and three species with E. Forest type C shared three species with D and three species with E. The *Gymnopus densilamellatus* Antonín, Ryoo & Ka, and *Suillus grevillei* (Klotzsch) Singer only appeared in E and C. The *S. luteus* (L.) Roussel only appeared in D and C; *Helvella crispa* (Scop.) Fr. only appeared in B and E. The *Amanita oberwinklerana* Zhu L. Yang & Yoshim., *Amanita orsonii* Ash. Kumar & T.N. Lakh., *Amanita virosa* Bertill., *Boletus edulis* Bull., *Phellinus pomaceus* (Pers.) Maire, *Russula paludosa* Britzelm., and *Tricholoma sejunctum* (Sowerby) Quél., only appeared in A and D. The *Agaricus moelleri* Wasser, *Gymnopus dryophilus* (Bull.) Murrill, *Pluteus leoninus* (Schaeff.) P. Kumm., and *Rhodocollybia butyracea* (Bull.) Lennox only appeared in A and C.

The species richness increased from E < C < D < B < A (Table 4). Broad-leaved forests A and B, with the highest richness indices of 7.4023 and 5.4832, respectively, accounted for 80.57% of the total species. Among them, 142 species were found in forest type A, accounting for 50.18% of the total species. This indicates that the broadleaf forest was the main habitat of macrofungi in the area, especially regarding the *Q. mongolica* forest. Mixed forests C and D, with richness indices of 2.8296 and 4.6509, contained 84 species, accounting for 29.68% of the total species. However, we found that the species abundance was higher in forest type D (49 species) than in forest type C (35 species), indicating that macrofungal species are associated with *Q. mongolica*. In *P. koraiensis* forest E, with the smallest species richness index of 2.286, we only found 18 species, accounting for 6.36% of the total species. This indicates that *P. koraiensis* forests can only provide habitats for a few fungal species.

### 3.6. Functional Diversity of Macrofungal Communities

The main functional groups were ectomycorrhizal fungi (EM), wood-decaying fungi (WS), and soil saprotroph (SS). The EM (133 species, 47.0%), WS (57 species, 20.14%), SS (52 species, 18.37%), and LS (38 species, 13.43%) increased from coniferous forest (E) < mixed coniferous forest (C, D) < broadleaf forest (A, B). EM fungi were most abundant in forest type A (Table 5). The highest content of EM fungi was *Amanita*, *Cortinarius*, *Lactarius,* and *Russula*. The most common WS were *Pleurotus* and *Polyporus*. The highest occurrence of SS was *Agaricus*, *Entoloma,* and *Hygrocybe*. The LS, *Clitocybe*, *Gymnopus*, *Mycena,* and *Pluteus*, showed the highest occurrence.

### 3.7. CCA Analysis of Macrofungal Communities and Selected Environmental Factors

**Figure 7:**
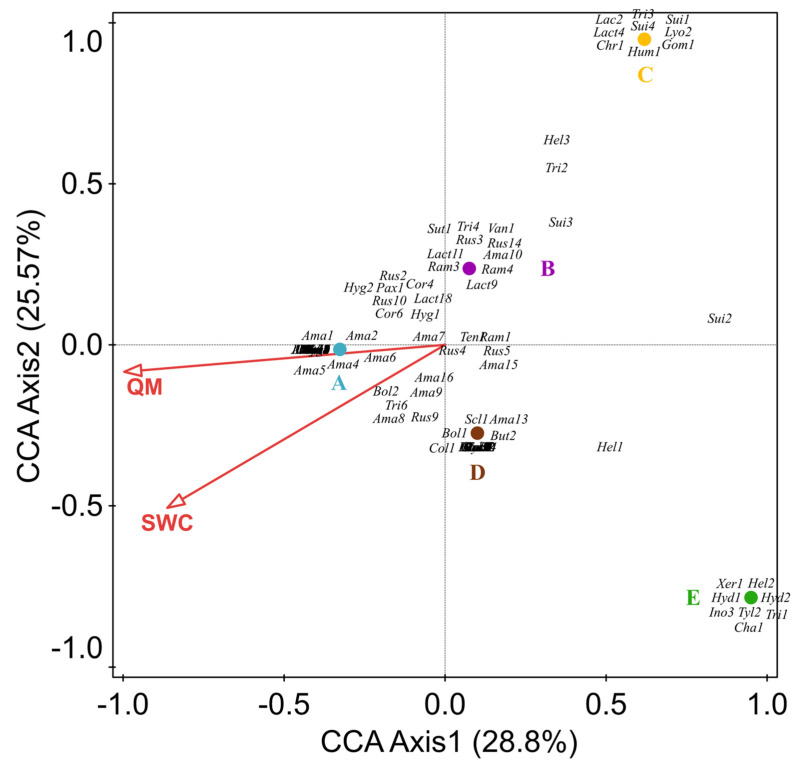
Canonical correspondence analysis (CCA) of selected variables and the ectomycorrhizal fungi species (dominant group). All displayed variables passed the most significant test (*p* < 0.05); QM: the number of *Q. mongolica*; SWC: soil water content; (**A**): *Q. mongolica* forest; (**B**): Broad–leaved forest; (**C**): *P. koraiensis* and *Q. mongolica* mixed forest with *P. koraiensis* being the dominant species; (**D**): *P. koraiensis* and *Q. mongolica* mixed forest with *Q. mongolica* being the dominant species; (**E**): *P. koraiensis* forest. Letters are composed of the first three–letter abbreviations of the scientific name of the species and a number, and the corresponding names are provided in Appendix C. Some species’ labels are overlapping. See Appendix C.

We performed a canonical correspondence analysis (CCA) for the 130 ectomycorrhizal fungi (EM) species recorded in the 5 forest types. The variables included *Q. mongolica* content, effective soil nitrogen, soil available phosphorus, soil available potassium, soil organic matter, soil pH, soil temperature, air temperature, soil water content, effective soil nitrogen, and air relative humidity. The CCA results show that all samples were roughly separated into five groups according to their corresponding locations. Eigenvalue axis 1 (0.8963) is higher than axis 2 (0.7955), with a cumulative contribution of 28.8% and 25.57%, respectively. Of all the variables, the *Q. mongolica* content and soil moisture content were the most significant factors influencing the EM fungi. Many EM fungi (e.g., *Amanita*, *Cortinarius*, *Lactarius*) positively correlate with *Q. mongolica* and soil water (Figure 7).

## 4. Discussion

This study is the first systematic survey of macrofungal diversity in Wunvfeng National Forest Park, Ji’an, China. We divided the forests into five main types: *Q. mongolica* forests (A), mixed broad-leaved forests (B), artificial *P. koraiensis* forests (E), and mixed forests (C, D). This enabled us to analyze the composition of macrofungi according to the relative content change of *Q. mongolica* in different forest types. The results show differences in species richness and diversity among forest types with different relative contents of *Q. mongolica*. The species richness increases with the relative content of *Q. mongolica*. Forest types with a high cover of *Q. mongolica* may provide a stable environment for the growth of macrofungi [47]. More importantly, *Quercus* is the main host plant of EM fungi [48], such as *Lactarius* [49,50], *Amanita* [51], *Russula* [52], and *Cortinarius* [53]. Our results reveal that the EM fungi are mainly distributed in the *Q. mongolica* forest. Most EM fungi had a significant positive correlation with *Q. mongolica* content (Figure 7), especially *Amanita*, *Cortinarius*, and *Lactarius*. In addition, we found that 11 species are shared in forest types A and D (e.g., *Amanita ibotengutake* T. Oda, C. Tanaka & Tsuda, *Amanita oberwinklerana* Zhu L. Yang & Yoshim, *Amanita orsonii* Ash. Kumar & T.N. Lakh., and *Amanita virosa* Bertill). However, they are not found in the *P. koraiensis* forest. These species may be associated with *Q. mongolica* (Figure 7, Ama7, Ama8, Ama9, Ama16), because the macrofungal communities change accordingly with the forest’s succession [54]. Therefore, these macrofungi shared in forest types D and A are likely to be in the early stages that develop from spore banks present in the soil [55].

The species richness of *P. koraiensis* forests is the lowest in our study. We only found 18 species. Theoretically, species richness may be similar between *Q. mongolica* and *P. koraiensis* forests because the EM fungi in temperate forests are significantly associated with *Quercus* and *Pinus* [56]. However, we only observed ten species of EM fungi in *P. koraiensis* forest (E) (e.g., *Hydnellum aurantiacum* (Batsch) P. Karst., *Hydnellum peckii* Banker, and *Tricholoma matsutake* (S. Ito & S. Imai) Singer). Our results differ from those of Gao [57], who found more EM fungi in *P. koraiensis* forests (aged < 150 years). On the one hand, EM fungi may need more time to form a stable symbiosis with the host plants [58]; on the other hand, the exotic trees have difficulty developing long-lasting symbiotic relationships with local EM fungi [59].

In our study, wood-dwelling fungi (57 species) are also a critical taxon that is mainly distributed in the *Q. mongolica* and mixed broad-leaved forests and grows on larger diameter *Q. mongolica* fallen wood (e.g., *Armillaria gallica* Marxm. & Romagn. and *Neolentinus cyathiformis* (Schaeff.) Della Magg. & Trassin). The wood-dwelling macrofungi may be related to forest type, as they tend to favor specific forest types under similar climatic conditions. In general, these combinations are determined by fungi closely related to the dominant tree, mainly because their enzymes have adapted to wood with different chemical and physical properties [60]. Another reason may be that large logs that provide a larger surface area have a greater chance of being colonized by fungal spores and mycelium than small logs. Species that produce large fruiting bodies also require more space [61]. Furthermore, we only found a few fallen trees in the *Q. mongolica* and mixed broadleaved forests; we found no fallen trees in the *P. koraiensis* forest, which is another factor that might affect fungal assemblage. The amount of deadwood also affects the macrofungal assemblage, which previous authors highlighted as the most crucial microhabitat in the forest [62,63]. The diversity of woody macrofungi strongly depends on the presence and amount of deadwood [64,65].

Saprophytic soil fungi (52 species) rely mainly on the decomposition of soil organic matter for nutrients, and they tend to prefer specific forest types under similar climatic conditions. Generally, the deciduous leaves of broad-leaved trees are more conducive to soil organic matter accumulation than coniferous forests [66]. The forest types with high soil organic matter have more opportunities to be colonized by fungal spores and mycelium [67]. Moreover, we only found thicker deciduous leaves in the *Q. mongolica* and mixed broad-leaved forests, affecting the grass rot fungal assemblage because litter saprotroph fungi strongly depend on deciduous leaves’ presence and volume [68].

The composition of fungi is also influenced and constrained by soil environmental variables [69,70]. These include soil moisture [71], soil pH [72], soil nutrients [73] and soil total C [74]. We analyzed the correlation between the main functional groups (ectomycorrhizal fungi) and selected environmental factors. The results showed that most ectomycorrhizal fungi are closely related to soil water content, especially *Amanita*, *Cortinarius*, *Lactarius*, and *Russula* (Figure 7). This result suggests that specific fungal communities respond to soil parameters differently [75,76,77]. Previous studies have shown that soil moisture is one factor that regulates the composition of the ectomycorrhizal fungi community [78,79,80,81]. Hydraulic lift contributes to maintaining EM fungi roots’ integrity and viability of extraradical hyphae [82]; further, EM fungi take up water and organic and inorganic nutrients from the soil via the extraradical hyphae and translocate these to colonized tree roots, receiving carbohydrates from the host in return [83]. This may be an important reason why most ectomycorrhizal fungi prefer forest types with relatively high soil water content.

This study with three years of species data is a small contribution that allows us to understand the distribution of fungal species in forest types with the different covers of *Q. mongolica*. The Wunvfeng National Forest Park has a strict protection policy for animals and plants, including soil protection. Thus, our soil data (with permission) are from July to September 2020 only. Nevertheless, our results illuminate the potential links between community composition and environmental factors because the July–September 2020 species data include almost all our species.

## 5. Conclusions

The *Q. mongolica* forests we analyzed are rich in macrofungal species. Although our data are based on only three years of sampling, we conclude that, as *Q. mongolica* increases in the forest, the abundance and diversity of macrofungal taxa also increase. We also observed that most EM species favored forest types with high *Q. mongolica* content (e.g., *Amanita*, *Cortinarius*, *Lactarius*, and *Russula*), indicating that some EM fungal communities are closely associated with *Q. mongolica*. We call for further studies to support this claim. In addition, we have only found *Tricholoma matsutake* (S. Ito & S. Imai) Singer in *P. koraiensis* forests, which is classified as an endangered species and considered an ectomycorrhizal fungus that is closely associated with *Pinus* trees. Therefore, according to our research, maintaining *P. koraiensis* forests is beneficial for conserving endangered species. However, deliberate conservation of *Q. mongolica* forests would be more useful for maintaining the diversity of macrofungal communities. Whether *P. koraiensis* affects other fungal species will need to be monitored over 3–5 years.

## Figures and Tables

**Table 1:** *Quercus mongolica* content and selected environmental variables properties in five forest types.

Environment Parameters	Forest				
	A	B	C	D	E
N (mg/kg)	68.2 ^a^	56.59 ^b^	44.07 ^c^	46.65 ^c^	47.94 ^c^
P (mg/kg)	20.99 ^b^	24.37 ^a^	18.33 ^b^	14.99 ^c^	20.49 ^b^
K (mg/kg)	408.9 ^a^	372.73 ^ab^	325.44 ^b^	267.35 ^c^	264.69 ^c^
SOM (g/kg)	37.1 ^a^	16.76 ^c^	25.03 ^b^	39.69 ^a^	28.64 ^b^
Soil pH	5.48 ^c^	5.96 ^a^	5.69 ^b^	5.34 ^c^	5.75 ^b^
Temp1 (°C)	24 ^a^	21.48 ^b^	22.12 ^b^	22.07 ^b^	22.07 ^b^
Temp2 (°C)	19.1 ^a^	18.93 ^a^	20.14 ^a^	19.99 ^a^	19.34 ^a^
SWC (g/20 g)	6 ^a^	5.2 ^a^	3.8 ^b^	5.6 ^a^	4.8 ^ab^
RH	0.82 ^a^	0.83 ^a^	0.83 ^a^	0.83 ^a^	0.86 ^a^
QM	275 ^a^	179 ^b^	42 ^c^	183 ^b^	0 ^d^

Note: Abbreviations: N = soil effective nitrogen; P = soil available phosphorus; K = soil available potassium; SOM = soil organic matter; pH = soil pH; Temp1 = soil temperature; Temp2 = air temperature; SWC = soil water content; RH = air relative humidity; QM = Number of *Q. mongolica*; Different lowercase letters indicate significantly different QM and environment parameter values among five forest type (*p* < 0.05).

**Table 2:** Dominant families (≥10 species) of macrofungi in Wunvfeng National Forest Park.

Family	Number of Species	Percentage (%)
*Russulaceae*	36	12.72%
*Tricholomataceae*	21	7.42%
*Boletaceae*	19	6.71%
*Amanitaceae*	16	5.65%
*Cortinariaceae*	14	4.95%
*Hygrophoraceae*	11	3.89%
*Agaricaceae*	10	3.53%
*Hymenochaetaceae*	10	3.53%
*Meruliaceae*	10	3.53%
Total	147	51.94%

**Table 3:** Dominant genera (≥5 species) of macrofungi in Wunvfeng National Forest Park.

Genera	Number of Species	Percentage (%)
*Lactarius*	18	6.36%
*Amanita*	16	5.65%
*Russula*	16	5.65%
*Cortinarius*	12	4.24%
*Tricholoma*	9	3.18%
*Mycena*	8	2.83%
*Suillus*	7	2.47%
*Entoloma*	7	2.47%
*Agaricus*	6	2.12%
*Gymnopus*	6	2.12%
*Hygrocybe*	6	2.12%
*Pluteus*	6	2.12%
*Clitocybe*	5	1.77%
*Marasmius*	5	1.77%
*Ramaria*	5	1.77%
total	132	46.64%

**Table 4:** Diversity indices of macrofungi in five forest types.

Forest Type	Number of Species	Number of Collections	Richness Index	Diversity Index	Evenness Index
			R	D	E
A	142	368	7.4023	4.7296	0.9543
B	86	246	5.4832	4.207	0.9445
C	35	153	2.8296	3.2048	0.9014
D	49	111	4.6509	3.7074	0.9526
E	18	62	2.286	2.6146	0.9046

Note: Abbreviations: A = *Q. mongolica* forest; B = Broad-leaved forest; C = *P, koraiensis* and *Q. mongolica* mixed forest, with *P. koraiensis* as the dominant species; D = *P. koraiensis* and *Q. mongolica* mixed forest, with *Q. mongolica* as the dominant species; E = *P. koraiensis* forest. The number of collections is the cumulative number of fruiting bodies per species.

**Table 5:** Cumulative species richness of functional groups in five forest types.

Forest Type	Trophic Groups					
	EM (133)	WS (57)	SS (52)	LS (38)	EI (2)	DS (1)
A	71	26	25	20	1	0
B	27	28	18	11	0	1
C	13	3	9	10	0	0
D	36	5	2	6	0	0
E	10	2	2	4	0	0

Note: Abbreviations: EM = Ectomycorrhizal fungi; WS = Wood-decaying fungi; SS = Soil saprotroph; LS = Litter saprotroph; EI = Endophyte-insect pathogen; DS = Dung saprotroph. Values shown are the cumulative number of macrofungal functional groups in each forest type.

**Figure A1:**
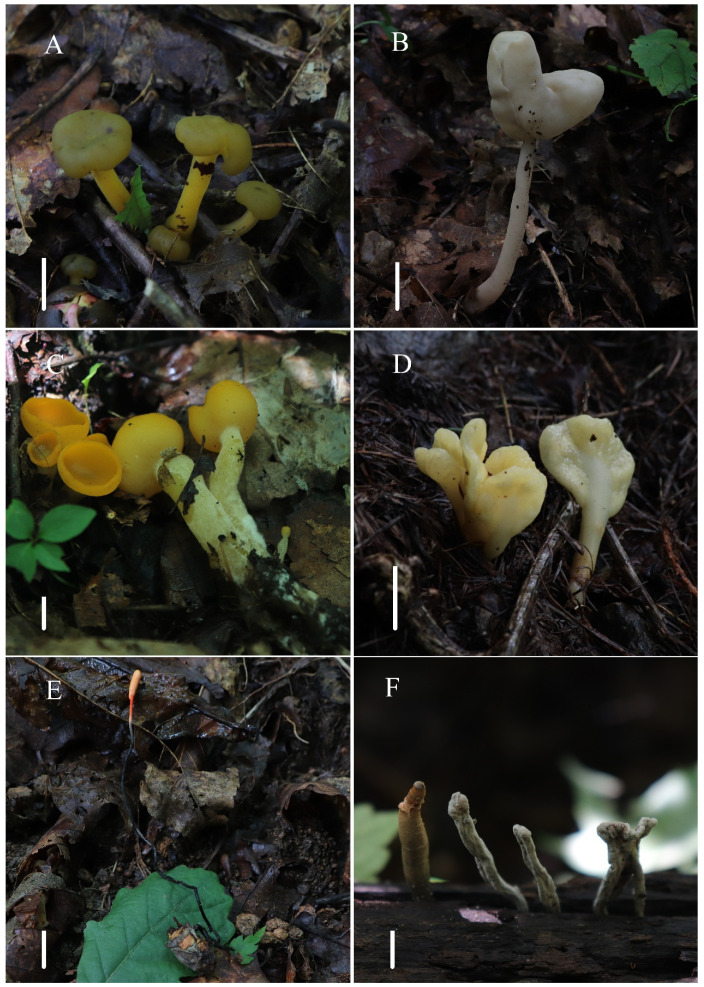
(**A**) *Leotia lubrica*; (**B**) *Helvella elastica*; (**C**) *Sowerbyella rhenana*; (**D**) *Spathularia flavida*; (**E**) *Ophiocordyceps nutans*; (**F**) *Xylaria hypoxylon*; Bars: 1 cm.

**Figure A2:**
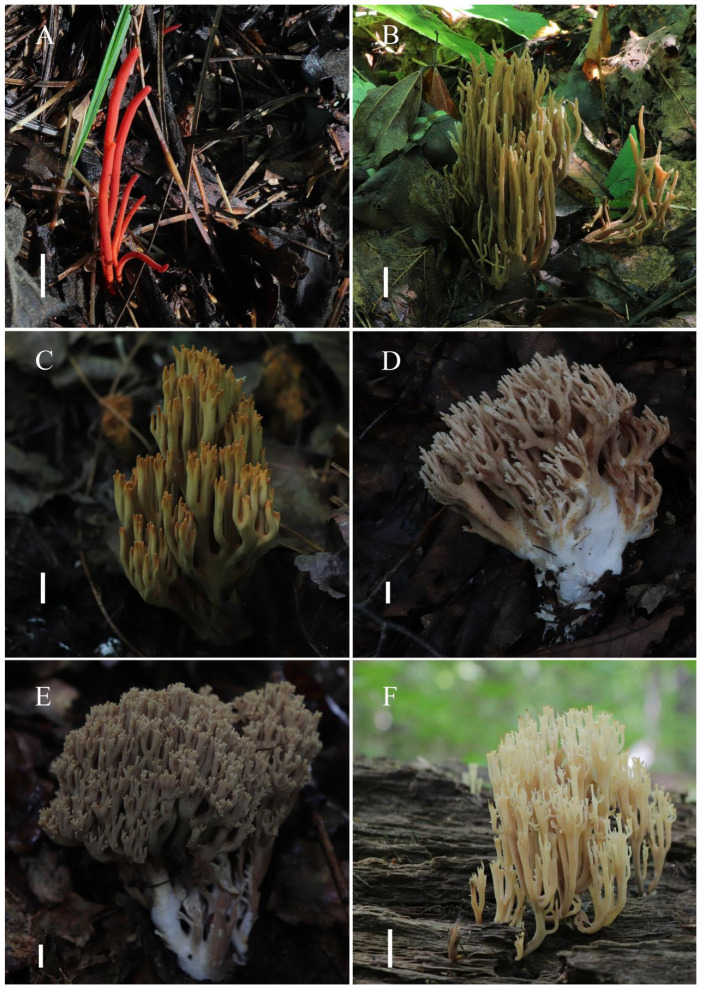
(**A**) *Clavulinopsis sulcata*; (**B**) *Ramaria stricta*; (**C**) *R. cokeri*; (**D**) *R. sanguinipes*; (**E**) *R. fennica*; (**F**) *Artomyces pyxidatus*; Bars: 1 cm.

**Figure A3:**
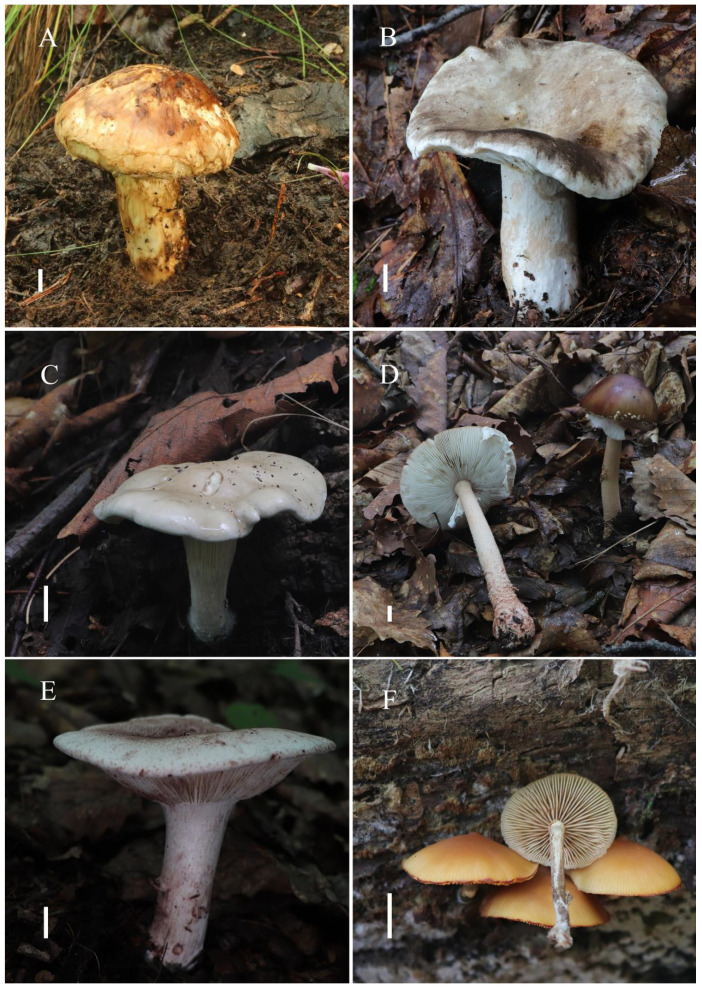
(**A**) *Tricholoma matsutake*; (**B**) *Russula furcata*; (**C**) *Clitocybe fasciculata*; (**D**) *Amanita orsonii*; (**E**) *Hygrophorus russula*; (**F**) *Galerina marginata*; Bars: 1 cm.

**Figure A4:**
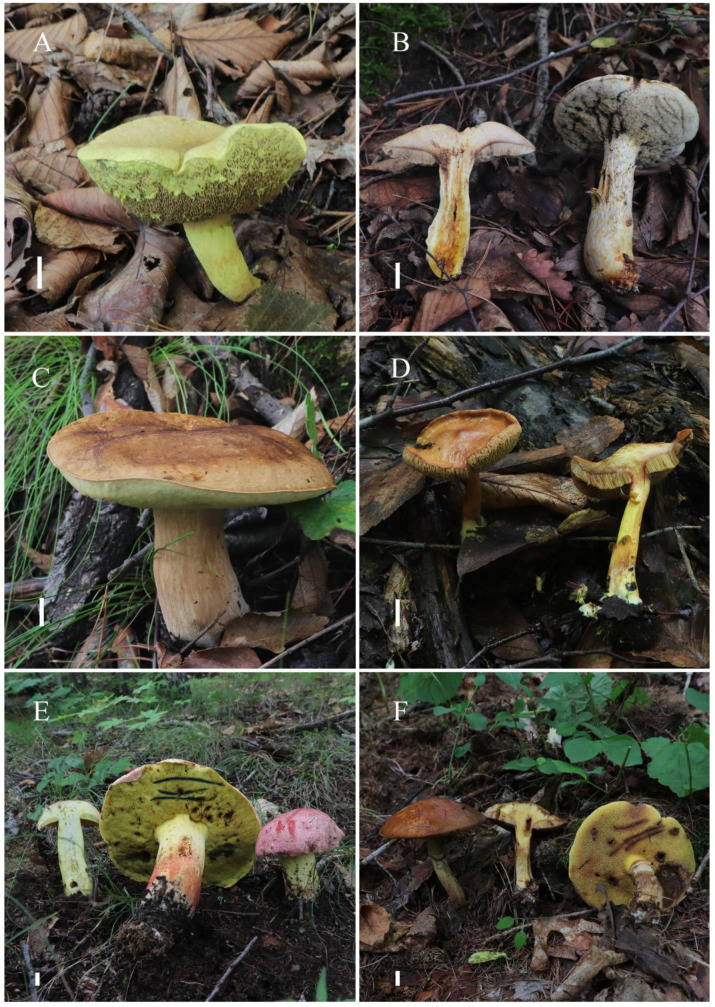
(**A**) *Pulveroboletus macrosporus*; (**B**) *Tylopilus felleus*; (**C**) *Boletus edulis*; (**D**) *Chalciporus piperatus*; (**E**) *Butyriboletus roseoflavus*; (**F**) *Suillus grevillei*; Bars: 1 cm.

**Figure A5:**
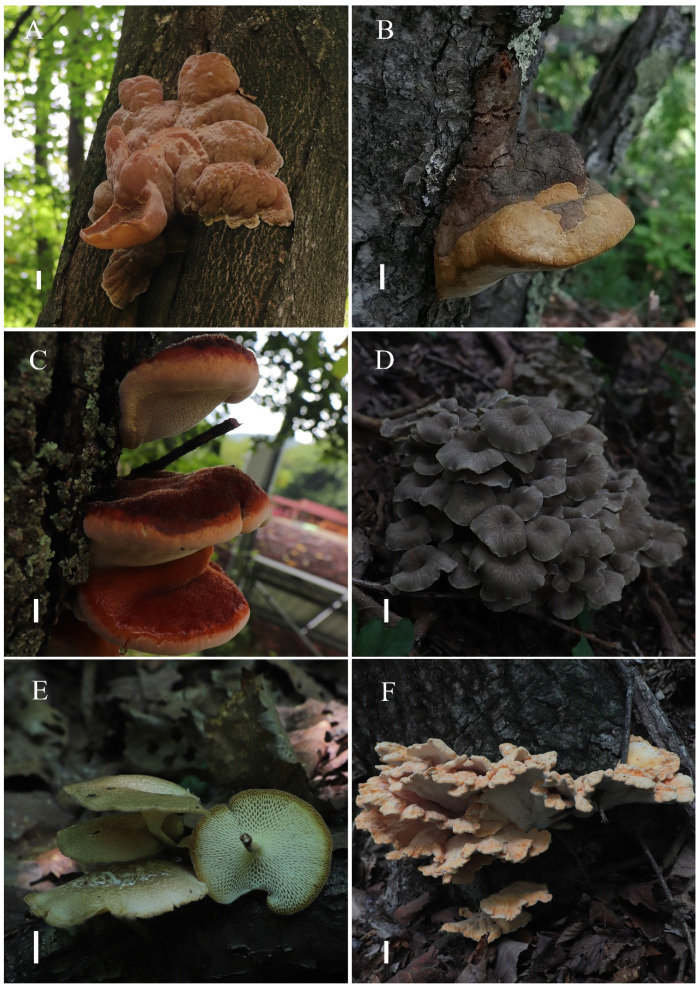
(**A**) *Gloeostereum incarnatum*; (**B**) *Phellinus pomaceus*; (**C**) *Inonotus hispidus*; (**D**) *Polyporus umbellatus*; (**E**) *Lentinus arcularius*; (**F**) *Laetiporus sulphureus*; Bars: 1 cm.

**Figure A6:**
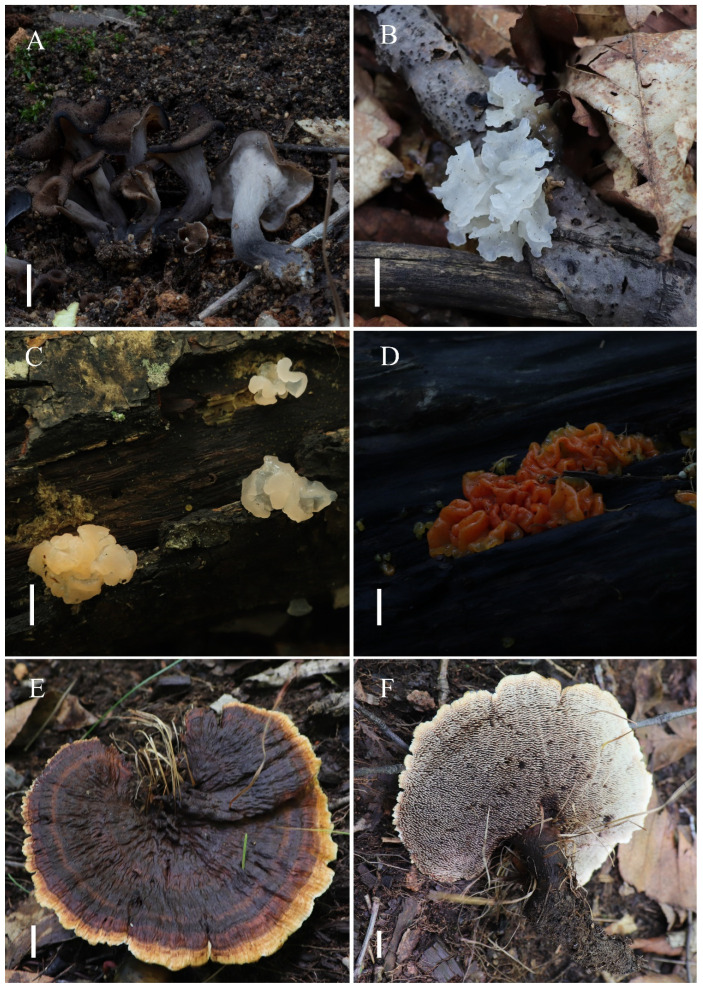
(**A**) *Craterellus cornucopioides*; (**B**) *Tremella fuciformis*; (**C**) *Dacrymyces yunnanensis*; (**D**) *D. chrysospermus*; (**E**,**F**) *Hydnellum aurantiacum*; Bars: 1 cm.

## Data Availability

Not applicable.

## Appendix A. Photos of Some Species

Larger Ascomycetes.

*Coral fungi.*

*Agarics.*

*Boletes.*

*Polyporoid fungi.*

*Jelly fungi* and *Hydnaceous fungi.*

## Appendix B. Macrofungi were collected in five forest types of Wunvfeng National Forest Park

**Species**	**Family**	**Genera**	**A**	**B**	**C**	**D**	**E**	**F**	**N**	**ML**	**Categories**	**SN**	**GenBank Number**
*Agaricus arvensis* Schaeff.	*Agaricaceae*	*Agaricus*	√						4	SS	Agarics	HMJAU60588	
*Agaricus dulcidulus* Schulzer	*Agaricaceae*	*Agaricus*	√						2	SS	Agarics	HMJAU60589	
*Agaricus moelleri* Wasser	*Agaricaceae*	*Agaricus*	√		√				3	SS	Agarics	HMJAU60590	
*Agaricus placomyces* Peck	*Agaricaceae*	*Agaricus*					√		1	SS	Agarics	HMJAU60591	
*Agaricus semotus* Fr.	*Agaricaceae*	*Agaricus*						√	2	SS	Agarics	HMJAU60592	
*Agaricus subrufescens* Peck	*Agaricaceae*	*Agaricus*	√						1	SS	Agarics	HMJAU60593	
*Agrocybe arvalis* (Fr.) Singer	*Strophariaceae*	*Agrocybe*		√					3	SS	Agarics	HMJAU60594	
*Agrocybe erebia* (Fr.) Kühner ex Singe	*Strophariaceae*	*Agrocybe*						√	2	SS	Agarics	HMJAU60595	
*Agrocybe praecox* (Pers.) Fayod	*Strophariaceae*	*Agrocybe*	√	√	√				6	SS	Agarics	HMJAU60596	
*Amanita altipes* Zhu L. Yang, M. Weiss & Oberw.	*Amanitaceae*	*Amanita*	√						1	EM	Agarics	HMJAU60597	
*Amanita chepangiana* Tulloss & Bhandary	*Amanitaceae*	*Amanita*	√						1	EM	Agarics	HMJAU60598	
*Amanita excelsa* (Fr.) Bertill.	*Amanitaceae*	*Amanita*				√			1	EM	Agarics	HMJAU60599	
*Amanita flavipes* S. Imai	*Amanitaceae*	*Amanita*	√						2	EM	Agarics	HMJAU60600	
*Amanita fuliginea* Hongo	*Amanitaceae*	*Amanita*	√						2	EM	Agarics	HMJAU60601	
*Amanita hemibapha* (Berk. & Broome) Sacc.	*Amanitaceae*	*Amanita*	√						2	EM	Agarics	HMJAU60602	
*Amanita ibotengutake* T. Oda, C. Tanaka & Tsuda	*Amanitaceae*	*Amanita*	√	√		√			6	EM	Agarics	HMJAU60603	
*Amanita oberwinklerana* Zhu L. Yang & Yoshim.	*Amanitaceae*	*Amanita*	√			√			3	EM	Agarics	HMJAU60604	
*Amanita orsonii* Ash. Kumar & T.N. Lakh.	*Amanitaceae*	*Amanita*	√			√			5	EM	Agarics	HMJAU60605	
*Amanita pallidocarnea* (Höhn.) Boedijn	*Amanitaceae*	*Amanita*		√					2	EM	Agarics	HMJAU60606	
*Amanita pallidorosea* P. Zhang & Zhu L. Yang	*Amanitaceae*	*Amanita*	√						2	EM	Agarics	HMJAU60607	
*Amanita rimosa* P. Zhang & Zhu L.	*Amanitaceae*	*Amanita*	√						1	EM	Agarics	HMJAU60608	
*Amanita rubescens* Pers.	*Amanitaceae*	*Amanita*				√			2	EM	Agarics	HMJAU60609	
*Amanita subglobosa* Zhu L. Yang	*Amanitaceae*	*Amanita*	√						1	EM	Agarics	HMJAU60610	
*Amanita vaginata* (Bull.) Lam.	*Amanitaceae*	*Amanita*		√		√			3	EM	Agarics	HMJAU60611	
*Amanita virosa* Secr.	*Amanitaceae*	*Amanita*	√			√			4	EM	Agarics	HMJAU60612	
*Ampulloclitocybe clavipes* (Pers.) Redhead, Lutzoni, Moncalvo & Vilgalys	*Hygrophoraceae*	*Ampulloclitocybe*	√						4	LS	Agarics	HMJAU60613	
*Armillaria gallica* Marxm. & Romagn.	*Physalacriaceae*	*Armillaria*	√	√		√			15	WS	Agarics	HMJAU60614	OL891486
*Armillaria mellea* (Vahl) P. Kumm.	*Physalacriaceae*	*Armillaria*	√						1	WS	Agarics	HMJAU60615	
*Artomyces pyxidatus* (Pers.) Jülich	*Auriscalpiaceae*	*Artomyces*	√	√					7	WS	Coral fungi	HMJAU60616	
*Bjerkandera adusta* (Willd.) P. Karst.	*Phanerochaetaceae*	*Bjerkandera*		√					1	WS	Polyporoid fungi	HMJAU60617	
*Boletus aereus* Bull.	*Boletaceae*	*Boletus*				√			2	EM	Boletes	HMJAU60618	
*Boletus edulis* Bull.	*Boletaceae*	*Boletus*	√			√			4	EM	Boletes	HMJAU60619	
*Bothia castanella* (Peck) Halling, T.J. Baroni & Manfr. Binder	*Boletaceae*	*Bothia*	√						2	EM	Boletes	HMJAU60620	
*Bulgaria inquinans* (Pers.) Fr.	*Phacidiaceae*	*Bulgaria*	√						1	WS	Larger Ascomytetes	HMJAU60621	
*Butyriboletus regius* D. Arora & J.L. Frank	*Boletaceae*	*Butyriboletus*	√						2	EM	Boletes	HMJAU60622	OL891490
*Butyriboletus roseoflavus* (Hai B. Li & Hai L. Wei) D. Arora & J.L. Frank	*Boletaceae*	*Butyriboletus*				√			3	EM	Boletes	HMJAU60623	OL891497
*Calocybe persicolor* (Fr.) Singer	*Lyophyllaceae*	*Calocybe*			√				3	SS	Agarics	HMJAU60624	
*Chalciporus piperatus* (Bull.) Bataille	*Boletaceae*	*Chalciporus*					√		1	EM	Boletes	HMJAU60625	
*Chroogomphus helveticus* (Singer) M.M. Moser	*Gomphidiaceae*	*Chroogomphus*			√				4	EM	Agarics	HMJAU60626	OL891485
*Clavaria fragilis* Holmsk.	*Clavariaceae*	*Clavaria*			√				1	SS	Coral fungi	HMJAU60627	
*Clavulinopsis corniculata* (Schaeff.) Corner	*Clavariaceae*	*Clavulinopsis*	√						1	SS	Coral fungi	HMJAU60628	
*Clavulinopsis sulcata* Overeem	*Clavariaceae*	*Clavulinopsis*					√		10	SS	Coral fungi	HMJAU60629	
*Clitocybe fasciculata* H.E. Bigelow & A.H. Sm.	*Tricholomataceae*	*Clitocybe*	√	√	√	√			2	LS	Agarics	HMJAU60630	
*Clitocybe geotropa* (Bull.) Quél.	*Tricholomataceae*	*Clitocybe*						√	1	LS	Agarics	HMJAU60631	
*Clitocybe gibba* (Pers.) P. Kumm.	*Tricholomataceae*	*Clitocybe*						√	4	LS	Agarics	HMJAU60632	
*Clitocybe odora* (Bull.) P. Kumm.	*Tricholomataceae*	*Clitocybe*	√						1	LS	Agarics	HMJAU60633	
*Clitocybe subditopoda* Peck	*Tricholomataceae*	*Clitocybe*	√	√			√		10	LS	Agarics	HMJAU60634	
*Coltricia crassa* Y.C. Dai	*Hymenochaetaceae*	*Coltricia*				√			2	EM	Polyporoid fungi	HMJAU60635	
*Coltricia strigosipes* Corner	*Hymenochaetaceae*	*Coltricia*	√						1	EM	Polyporoid fungi	HMJAU60636	
*Coprinellus disseminatus* (Pers.) J.E. Lange	*Psathyrellaceae*	*Coprinellus*		√					8	SS	Agarics	HMJAU60637	
*Coprinellus radians* (Desm.) Vilgalys, Hopple & Jacq. Johnson	*Psathyrellaceae*	*Coprinellus*	√						5	SS	Agarics	HMJAU60638	
*Coprinus comatus* (O.F. Müll.) Pers.	*Agaricaceae*	*Coprinus*		√					1	DS	Agarics	HMJAU60639	
*Cordyceps militaris Cordyceps militaris* (L.) *Fr.*	*Cordycipitaceae*	*Cordyceps*						√	3	EI	Larger Ascomytetes	HMJAU60640	
*Cortinarius anomalus* (Fr.) Fr.	*Cortinariaceae*	*Cortinarius*				√			2	EM	Agarics	HMJAU60641	OL891464
*Cortinarius armillatus* (Fr.) Fr.	*Cortinariaceae*	*Cortinarius*	√						4	EM	Agarics	HMJAU60642	OL891465
*Cortinarius balaustinus* Fr.	*Cortinariaceae*	*Cortinarius*	√						3	EM	Agarics	HMJAU60643	
*Cortinarius bivelus* (Fr.) Fr.	*Cortinariaceae*	*Cortinarius*	√	√					5	EM	Agarics	HMJAU60644	OL891467
*Cortinarius caperatus* (Pers.) Fr.	*Cortinariaceae*	*Cortinarius*	√						1	EM	Agarics	HMJAU60645	OL891466
*Cortinarius cotoneus* Fr.	*Cortinariaceae*	*Cortinarius*	√	√					3	EM	Agarics	HMJAU60646	OL891468
*Cortinarius ectypus* J. Favre	*Cortinariaceae*	*Cortinarius*	√						2	EM	Agarics	HMJAU60647	
*Cortinarius flammeouraceus* Niskanen, Kytov., Liimat., Ammirati & Dima	*Cortinariaceae*	*Cortinarius*	√						4	EM	Agarics	HMJAU60648	OL891470
*Cortinarius hesleri* Ammirati, Niskanen, Liimat. & Matheny	*Cortinariaceae*	*Cortinarius*	√						1	EM	Agarics	HMJAU60649	
*Cortinarius pholideus* (Lilj.) Fr.	*Cortinariaceae*	*Cortinarius*				√			3	EM	Agarics	HMJAU60650	OL891469
*Cortinarius sanguineus* (Wulfen) Gray	*Cortinariaceae*	*Cortinarius*				√			2	EM	Agarics	HMJAU60651	
*Cortinarius subbalaustinus* Rob. Henry	*Cortinariaceae*	*Cortinarius*	√						1	EM	Agarics	HMJAU60652	
*Cortinarius torvus* (Fr.) Fr.	*Cortinariaceae*	*Cortinarius*	√						1	EM	Agarics	HMJAU60653	
*Cotylidia diaphana* (Cooke) Lentz	*Rickenellaceae*	*Cotylidia*			√				4	SS	Jelly fungi	HMJAU60654	
*Craterellus cornucopioides* (L.) Pers.	*Hydnaceae*	*Craterellus*	√						1	EM	Cantharelloid	HMJAU60655	
*Crepidotus applanatus* (Pers.) P. Kumm.	*Crepidotaceae*	*Crepidotus*				√			1	WS	Agarics	HMJAU60656	
*Crepidotus malachius* Sacc.	*Crepidotaceae*	*Crepidotus*		√					5	WS	Agarics	HMJAU60657	
*Cyclocybe erebia* (Fr.) Vizzini & Matheny	*Tubariaceae*	*Cyclocybe*	√						2	SS	Agarics	HMJAU60658	
*Cystoderma granulosum* (Batsch) Fayod	*Agaricaceae*	*Cystoderma*	√						4	SS	Agarics	HMJAU60659	
*Dacrymyces chrysospermus Berk. & M.A. Curtis*	*Dacrymycetaceae*	*Dacrymyces*	√						3	WS	Jelly fungi	HMJAU60660	
*Dacrymyces yunnanensis* B. Liu & L. Fan	*Dacrymycetaceae*	*Dacrymyces*	√	√					6	WS	Jelly fungi	HMJAU60661	
*Daldinia concentrica* (Bolton) Ces. & De Not.	*Hypoxylaceae*	*Daldinia*		√					5	WS	Larger Ascomytetes	HMJAU60662	
*Elmerina cladophora* (Berk.) Bres.	*Auriculariaceae*	*Elmerina*	√	√					3	WS	Polyporoid fungi	HMJAU60663	
*Elmerina hispida* (Imazeki) Y.C. Dai & L.W. Zhou	*Auriculariaceae*	*Elmerina*				√			1	WS	Polyporoid fungi	HMJAU60664	
*Entoloma abortivum* (Berk. & M.A. Curtis) Donk	*Entolomataceae*	*Entoloma*		√					2	SS	Agarics	HMJAU60665	
*Entoloma alboumbonatum* Hesler	*Entolomataceae*	*Entoloma*				√			2	SS	Agarics	HMJAU60666	
*Entoloma caespitosum* W.M. Zhang	*Entolomataceae*	*Entoloma*		√					5	SS	Agarics	HMJAU60667	
*Entoloma clypeatum* (L.) P. Kumm.	*Entolomataceae*	*Entoloma*	√						4	SS	Agarics	HMJAU60668	
*Entoloma holoconiotum* (Largent & Thiers) Noordel. & Co-David	*Entolomataceae*	*Entoloma*		√					4	SS	Agarics	HMJAU60669	
*Entoloma mediterraneense* Noordel. & Hauskn.	*Entolomataceae*	*Entoloma*	√						1	SS	Agarics	HMJAU60670	
*Entoloma rhodopolium* (Fr.) P. Kumm.	*Entolomataceae*	*Entoloma*	√						2	SS	Agarics	HMJAU60671	
*Entonaema liquescens Möller*	*Hypoxylaceae*	*Entonaema*		√					3	WS	Larger Ascomytetes	HMJAU60672	
*Favolus squamosus* (Huds.) A. Ames	*Polyporaceae*	*Favolus*	√						1	WS	Polyporoid fungi	HMJAU60673	
*Flammulaster erinaceellus* (Peck) Watling	*Tubariaceae*	*Flammulaster*		√					6	WS	Agarics	HMJAU60674	
*Galerina helvoliceps* (Berk. & M.A. Curtis) Singe	*Hymenogastraceae*	*Galerina*	√						4	WS	Agarics	HMJAU60675	
*Galerina marginata* (Batsch) Kühner	*Hymenogastraceae*	*Galerina*		√					4	WS	Agarics	HMJAU60676	
*Geastrum triplex* Jungh.	*Geastraceae*	*Geastrum*	√						1	LS	Gasteroid fungi	HMJAU60677	
*Gerhardtia borealis* (Fr.) Contu & A. Ortega	*Lyophyllaceae*	*Gerhardtia*				√			3	SS	Agarics	HMJAU60678	
*Gerronema nemorale* Har. Takah.	*Marasmiaceae*	*Gerronema*	√						1	WS	Agarics	HMJAU60679	
*Gloeostereum incarnatum* S. Ito & S. Imai	*Cyphellaceae*	*Gloeostereum*		√					4	WS	Polyporoid fungi	HMJAU60680	
*Gomphidius maculatus* (Scop.) Fr.	*Gomphidiaceae*	*Gomphidius*			√				2	EM	Cantharelloid	HMJAU60681	
*Gymnopilus junonius* (Fr.) P.D. Orton	*Hymenogastraceae*	*Gymnopilus*		√					3	WS	Agarics	HMJAU60682	
*Gymnopilus penetrans* (Fr.) Murrill	*Hymenogastraceae*	*Gymnopilus*		√					2	WS	Agarics	HMJAU60683	
*Gymnopilus suberis* (Maire) Singer	*Hymenogastraceae*	*Gymnopilus*		√					6	WS	Agarics	HMJAU60684	
*Gymnopus alnicola* J.L. Mata & Halling	*Omphalotaceae*	*Gymnopus*			√				4	LS	Agarics	HMJAU60685	
*Gymnopus confluens* (Pers.) Antonín, Halling & Noordel.	*Omphalotaceae*	*Gymnopus*			√		√		14	LS	Agarics	HMJAU60686	OL884222
*Gymnopus densilamellatus* Antonín, Ryoo & Ka	*Omphalotaceae*	*Gymnopus*	√	√	√		√		25	LS	Agarics	HMJAU60687	OL884223
*Gymnopus dryophilus* (Bull.) Murrill	*Omphalotaceae*	*Gymnopus*	√		√				30	LS	Agarics	HMJAU60688	OL891463
*Gymnopus gibbosus* (Corner) A.W. Wilson, Desjardin & E. Horak	*Omphalotaceae*	*Gymnopus*					√		7	LS	Agarics	HMJAU60689	OL884224
*Gymnopus loiseleurietorum* (M.M. Moser, Gerhold & Tobies) Antonín & Noordel.	*Omphalotaceae*	*Gymnopus*	√						7	LS	Agarics	HMJAU60690	
*Harrya chromapes* (Frost) Halling, Nuhn, Osmundson & Manfr.	*Boletaceae*	*Harrya*	√						1	EM	Boletes	HMJAU60691	OL891492
*Hebeloma birrus* (Fr.) Gillet	*Hymenogastraceae*	*Hebeloma*	√						6	EM	Agarics	HMJAU60692	OL891483
*Heliocybe sulcata* (Berk.) Redhead & Ginns	*Gloeophyllaceae*	*Heliocybe*	√						2	WS	Agarics	HMJAU60693	
*Helvella crispa* (Scop.) Fr.	*Hydnaceae*	*Helvella*		√			√		2	EM	Larger Ascomytetes	HMJAU60694	
*Helvella elastica* Bull.	*Helvellaceae*	*Helvella*					√		1	EM	Larger Ascomytetes	HMJAU60695	
*Helvella macropus* (Pers.) P. Karst.	*Helvellaceae*	*Helvella*		√	√				3	EM	Larger Ascomytetes	HMJAU60696	
*Hemistropharia albocrenulata* (Peck) Jacobsson & E. Larss.	*Tubariaceae*	*Hemistropharia*		√					1	SS	Agarics	HMJAU60697	
*Hericium erinaceus (Bull.) Pers.*	*Hericiaceae*	*Hericium*		√					1	WS	Hydnaceous fungi	HMJAU60698	
*Humaria hemisphaerica* (F.H. Wigg.) Fuckel	*Pyronemataceae*	*Humaria*			√				1	EM	Larger Ascomytetes	HMJAU60699	
*Hydnellum aurantiacum* (Batsch) P. Karst.	*Bankeraceae*	*Hydnellum*					√		2	EM	Hydnaceous fungi	HMJAU60700	OL891471
*Hydnellum peckii* Banker	*Bankeraceae*	*Hydnellum*					√		2	EM	Hydnaceous fungi	HMJAU60701	OL891487
*Hygrocybe cantharellus* (Schwein.) Murrill	*Hygrophoraceae*	*Hygrocybe*	√						5	SS	Agarics	HMJAU60702	
*Hygrocybe chlorophana* (Fr.) Wünsche	*Hygrophoraceae*	*Hygrocybe*		√					5	SS	Agarics	HMJAU60703	
*Hygrocybe coccinea* (Schaeff.) P. Kumm.	*Hygrophoraceae*	*Hygrocybe*	√						4	SS	Agarics	HMJAU60704	
*Hygrocybe coccineocrenata*.D. Orton) M.M. Moser	*Hygrophoraceae*	*Hygrocybe*		√					5	SS	Agarics	HMJAU60705	
*Hygrocybe miniata* (Fr.) P. Kumm.	*Hygrophoraceae*	*Hygrocybe*	√						4	SS	Agarics	HMJAU60706	
*Hygrocybe reidii* Kühner	*Hygrophoraceae*	*Hygrocybe*		√					1	SS	Agarics	HMJAU60707	
*Hygrophoropsis aurantiaca* (Wulfen) Maire	*Hygrophoropsidaceae*	*Hygrophoropsis*	√	√					7	LS	Agarics	HMJAU60708	OL891484
*Hygrophorus conicus* (Schaeff.) Fr.	*Hygrophoraceae*	*Hygrophorus*	√	√					6	EM	Agarics	HMJAU60709	
*Hygrophorus nemoreus* (Pers.) Fr.	*Hygrophoraceae*	*Hygrophorus*	√	√					9	EM	Agarics	HMJAU60710	
*Hygrophorus pudorinus* (Fr.) Fr.	*Hygrophoraceae*	*Hygrophorus*	√						1	EM	Agarics	HMJAU60711	
*Hygrophorus russula* (Schaeff. ex Fr.) Kauffman	*Hygrophoraceae*	*Hygrophorus*	√						2	EM	Agarics	HMJAU60712	
*Hypholoma capnoides* (Fr.) P. Kumm.	*Strophariaceae*	*Hypholoma*					√		2	WS	Agarics	HMJAU60713	
*Hypholoma sublateritium* (Fr.) Quél.	*Strophariaceae*	*Hypholoma*	√						4	WS	Agarics	HMJAU60714	
*Infundibulicybe geotropa* (Bull.) Harmaja	*Tricholomataceae*	*Infundibulicybe*		√					2	SS	Agarics	HMJAU60715	
*Infundibulicybe gibba* (Pers.) Harmaja	*Tricholomataceae*	*Infundibulicybe*	√						2	SS	Agarics	HMJAU60716	
*Inocybe assimilata* Britzelm.	*Inocybaceae*	*Inocybe*	√						2	EM	Agarics	HMJAU60717	
*Inocybe asterospora* Quél.	*Inocybaceae*	*Inocybe*	√						1	EM	Agarics	HMJAU60718	
*Inocybe suaveolens* D.E. Stuntz	*Inocybaceae*	*Inocybe*					√		2	EM	Agarics	HMJAU60719	OL891472
*Inonotus hispidus* (Bull.) P. Karst.	*Hymenochaetaceae*	*Inonotus*		√					3	WS	Polyporoid fungi	HMJAU60720	
*Laccaria amethystina* Cooke	*Hydnangiaceae*	*Laccaria*				√			4	EM	Agarics	HMJAU60721	
*Laccaria laccata* (Scop.) Cooke	*Hydnangiaceae*	*Laccaria*			√				4	EM	Agarics	HMJAU60722	
*Lactarius albidocinereus* X.H. Wang, S.F. Shi & T. Bau	*Russulaceae*	*Lactarius*				√			2	EM	Agarics	HMJAU60723	OL891473
*Lactarius brunneoviolascens* Bon	*Russulaceae*	*Lactarius*	√						1	EM	Agarics	HMJAU60724	
*Lactarius conglutinatus* X.H. Wang	*Russulaceae*	*Lactarius*	√						6	EM	Agarics	HMJAU60725	
*Lactarius deterrimus* Gröger	*Russulaceae*	*Lactarius*			√				5	EM	Agarics	HMJAU60726	
*Lactarius flavidus* Boud.	*Russulaceae*	*Lactarius*	√						1	EM	Agarics	HMJAU60727	
*Lactarius glyciosmus* (Fr.) Fr.	*Russulaceae*	*Lactarius*	√						1	EM	Agarics	HMJAU60728	
*Lactarius hirtipes* J.Z. Ying	*Russulaceae*	*Lactarius*	√						3	EM	Agarics	HMJAU60729	
*Lactarius lilacinus* Fr.	*Russulaceae*	*Lactarius*	√						3	EM	Agarics	HMJAU60730	
*Lactarius pallidus* Pers.	*Russulaceae*	*Lactarius*		√					2	EM	Agarics	HMJAU60731	
*Lactarius piperatus* (L.) Pers.	*Russulaceae*	*Lactarius*	√						1	EM	Agarics	HMJAU60732	
*Lactarius proximellus* Beardslee & Burl.	*Russulaceae*	*Lactarius*		√					1	EM	Agarics	HMJAU60733	
*Lactarius pubescens* Fr.	*Russulaceae*	*Lactarius*	√						1	EM	Agarics	HMJAU60734	
*Lactarius subvellereus* Peck	*Russulaceae*	*Lactarius*	√						2	EM	Agarics	HMJAU60735	
*Lactarius torminosus* (Schaeff.) Pers.	*Russulaceae*	*Lactarius*				√			2	EM	Agarics	HMJAU60736	
*Lactarius trivialis* (Fr.) Fr.	*Russulaceae*	*Lactarius*	√						1	EM	Agarics	HMJAU60737	OL891474
*Lactarius vietus* (Fr.) Fr.	*Russulaceae*	*Lactarius*	√						2	EM	Agarics	HMJAU60738	
*Lactarius volemus* (Fr.) Fr.	*Russulaceae*	*Lactarius*	√						3	EM	Agarics	HMJAU60739	
*Lactarius zonarius* (Bull.) Fr.	*Russulaceae*	*Lactarius*	√	√					2	EM	Agarics	HMJAU60740	
*Lactifluus bertillonii* (Neuhoff ex Z. Schaef.) Verbeken	*Russulaceae*	*Lactifluus*	√						1	EM	Agarics	HMJAU60741	OL891489
*Lactifluus pilosus* (Verbeken, H.T. Le & Lumyong) Verbeken	*Russulaceae*	*Lactifluus*	√						1	EM	Agarics	HMJAU60742	
*Laetiporus sulphureus* (Bull.) Murrill	*Laetiporaceae*	*Laetiporus*	√	√					2	WS	Polyporoid fungi	HMJAU60743	
*Leccinum aurantiacum* (Bull.) Gray	*Boletaceae*	*Leccinum*	√						2	EM	Boletes	HMJAU60744	
*Leccinum scabrum* (Bull.) Gray	*Boletaceae*	*Leccinum*				√			1	EM	Boletes	HMJAU60745	
*Leccinum versipelle* (Fr. & Hök) Snell	*Boletaceae*	*Leccinum*	√						1	EM	Boletes	HMJAU60746	
*Lentinellus ursinus* (Fr.) Kühner	*Auriscalpiaceae*	*Lentinellus*	√						6	WS	Agarics	HMJAU60747	
*Lentinus edodes* (Berk.) Singer	*Omphalotaceae*	*Lentinus*	√						2	WS	Agarics	HMJAU60748	
*Lentinus sajor-caju* (Fr.) Fr.	*Polyporaceae*	*Lentinus*		√					6	WS	Agarics	HMJAU60749	OL891475
*Leotia lubrica* (Scop.) Pers.	*Leotiaceae*	*Leotia*	√						4	SS	Larger Ascomytetes	HMJAU60750	
*Lepiota castanea* Quél.	*Agaricaceae*	*Lepiota*	√						5	LS	Agarics	HMJAU60751	
*Lepiota cristata* (Bolton) P. Kumm.	*Agaricaceae*	*Lepiota*	√						4	LS	Agarics	HMJAU60752	
*Lepista nuda* (Bull.) Cooke	*Tricholomataceae*	*Lepista*	√	√					9	SS	Agarics	HMJAU60753	
*Lepista personata* (Fr.) Cooke	*Tricholomataceae*	*Lepista*		√					1	SS	Agarics	HMJAU60754	
*Leucocybe connata* (Schumach.) Vizzini, P. Alvarado, G. Moreno & Consiglio	*Lyophyllaceae*	*Leucocybe*	√						3	EM	Agarics	HMJAU60755	
*Lycoperdon mammiforme* Pers.	*Lycoperdaceae*	*Lycoperdon*	√						8	SS	Agarics	HMJAU60756	
*Lycoperdon perlatum* Pers.	*Lycoperdaceae*	*Lycoperdon*	√	√					5	SS	Gasteroid fungi	HMJAU60757	
*Lycoperdon umbrinum* Pers.	*Lycoperdaceae*	*Lycoperdon*		√					3	SS	Gasteroid fungi	HMJAU60758	
*Lyophyllum decastes* (Fr.) Singer	*Lyophyllaceae*	*Lyophyllum*	√						3	EM	Agarics	HMJAU60759	
*Lyophyllum infumatum* (Bres.) Kühner	*Lyophyllaceae*	*Lyophyllum*			√				1	EM	Agarics	HMJAU60760	OL891476
*Macrocystidia cucumis* (Pers.) Joss.	*Macrocystidiaceae*	*Macrocystidia*		√					2	SS	Agarics	HMJAU60761	
*Marasmius confertus* Berk. & Broome	*Marasmiaceae*	*Marasmius*	√						2	LS	Agarics	HMJAU60762	
*Marasmius maximus* Hongo	*Marasmiaceae*	*Marasmius*	√	√					8	LS	Agarics	HMJAU60763	
*Marasmius nigrodiscus* (Peck) Halling	*Marasmiaceae*	*Marasmius*			√				2	LS	Agarics	HMJAU60764	
*Marasmius occultatiformis* Antonín, Ryoo & H.D. Shin	*Marasmiaceae*	*Marasmius*				√			4	LS	Agarics	HMJAU60765	
*Marasmius siccus* (Schwein.) Fr.	*Marasmiaceae*	*Marasmius*		√					1	LS	Agarics	HMJAU60766	
*Megacollybia clitocyboidea* R.H. Petersen, Takehashi & Nagas.	*Megacollybia*	*Megacollybia*	√	√					4	WS	Agarics	HMJAU60767	
*Melanoleuca cognata* (Huds.) Fr.	*Tricholomataceae*	*Melanoleuca*			√				1	SS	Agarics	HMJAU60768	
*Mucidula brunneomarginata* Lj.N. Vassiljeva) R.H. Petersen	*Physalacriaceae*	*Mucidula*		√					2	WS	Agarics	HMJAU60769	
*Mutinus caninus* (Huds.) Fr.	*Phallaceae*	*Mutinus*			√				4	LS	Gasteroid fungi	HMJAU60770	
*Mycena galericulata* (Scop.) Gray	*Mycenaceae*	*Mycena*				√			5	LS	Agarics	HMJAU60771	
*Mycena haematopus* (Pers.) P. Kumm.	*Mycenaceae*	*Mycena*			√				3	LS	Agarics	HMJAU60772	
*Mycena pelianthina* (Fr.) Quél.	*Mycenaceae*	*Mycena*						√	1	LS	Agarics	HMJAU60773	
*Mycena polygramma* (Bull.) Gray	*Mycenaceae*	*Mycena*				√			2	LS	Agarics	HMJAU60774	
*Mycena pura* (Pers.) P. Kumm.	*Mycenaceae*	*Mycena*		√	√	√			4	LS	Agarics	HMJAU60775	
*Mycena roseocandida* (Peck) Sacc.	*Mycenaceae*	*Mycena*	√						1	LS	Agarics	HMJAU60776	
*Mycena sanguinolenta* (Alb. & Schwein.) P. Kumm.	*Mycenaceae*	*Mycena*	√						8	LS	Agarics	HMJAU60777	
*Mycena stylobates* (Pers.) P. Kumm.	*Mycenaceae*	*Mycena*		√		√			12	LS	Agarics	HMJAU60778	
*Neolentinus cyathiformis* (Schaeff.) Della Magg. & Trassin.	*Gloeophyllaceae*	*Neolentinus*	√	√					15	WS	Agarics	HMJAU60779	OL891477
*Omphalina pyxidata* (Bull.) Quél.	*Tricholomataceae*	*Omphalina*		√					3	LS	Agarics	HMJAU60780	
*Ophiocordyceps nutans* (Pat.) G.H. Sung, J.M. Sung, Hywel-Jones & Spatafora	*Ophiocordycipitaceae*	*Ophiocordyceps*		√					1	EI	Larger Ascomytetes	HMJAU60781	
*Oudemansiella mucida* (Schrad.) Höhn.	*Physalacriaceae*	*Oudemansiella*	√						4	WS	Agarics	HMJAU60782	
*Paxillus involutus* (Batsch) Fr.	*Paxillaceae*	*Paxillus*	√	√					2	EM	Agarics	HMJAU60783	
*Paxillus orientalis* Gelardi, Vizzini, E. Horak & G. Wu	*Paxillaceae*	*Paxillus*	√						1	EM	Agarics	HMJAU60784	
*Peziza domiciliana* Cooke	*Pezizaceae*	*Peziza*	√						3	SS	Larger Ascomytetes	HMJAU60785	
*Phallus tenuissimus* T.H. Li, W.Q. Deng & B. Liu	*Phallaceae*	*Phallus*		√					2	LS	Gasteroid fungi	HMJAU60786	
*Phellinus pomaceus* (Pers.) Maire	*Hymenochaetaceae*	*Phellinus*	√			√			2	WS	Polyporoid fungi	HMJAU60787	
*Pholiota aurivella* (Batsch) P. Kumm.	*Strophariaceae*	*Pholiota*	√						4	WS	Agarics	HMJAU60788	
*Pholiota squarrosoides* (Peck) Sacc.	*Strophariaceae*	*Pholiota*						√	3	WS	Agarics	HMJAU60789	
*Phylloporus yunnanensis* N.K. Zeng, Zhu L. Yang & L.P. Tang	*Boletaceae*	*Phylloporus*	√						2	EM	Boletes	HMJAU60790	
*Phyllotopsis nidulans* (Pers.) Singer	*Phyllotopsidaceae*	*Phyllotopsis*			√				7	WS	Agarics	HMJAU60791	
*Piptoporus soloniensis* (Dubois) Pilát	*Fomitopsidaceae*	*Piptoporus*						√	2	WS	Polyporoid fungi	HMJAU60792	OL891488
*Pleurotus citrinopileatus* Singer	*Pleurotaceae*	*Pleurotus*		√					4	WS	Agarics	HMJAU60793	
*Pleurotus ostreatus* (Jacq.) P. Kumm.	*Pleurotaceae*	*Pleurotus*	√	√					8	WS	Agarics	HMJAU60794	OL891478
*Pleurotus pulmonarius* (Fr.) Quél.	*Pleurotaceae*	*Pleurotus*	√						2	WS	Agarics	HMJAU60795	
*Pluteus atromarginatus* (Konrad) Kühner	*Pluteaceae*	*Pluteus*		√					2	LS	Agarics	HMJAU60796	
*Pluteus cervinus* (Schaeff.) P. Kumm.	*Pluteaceae*	*Pluteus*	√						1	LS	Agarics	HMJAU60797	
*Pluteus hispidulus* (Fr.) Gillet	*Pluteaceae*	*Pluteus*	√						1	LS	Agarics	HMJAU60798	
*Pluteus leoninus* (Schaeff.) P. Kumm	*Pluteaceae*	*Pluteus*	√		√				1	LS	Agarics	HMJAU60799	
*Pluteus phlebophorus* (Ditmar) P. Kumm.	*Pluteaceae*	*Pluteus*	√						2	LS	Agarics	HMJAU60800	
*Pluteus pouzarianus* Singer	*Pluteaceae*	*Pluteus*	√						2	LS	Agarics	HMJAU60801	
*Podoscypha nitidula* (Berk.) Pat.	*Podoscyphaceae*	*Podoscypha*		√					3	WS	Polyporoid fungi	HMJAU60802	
*Polyporus arcularius* (Batsch) Fr.	*Polyporaceae*	*Polyporus*		√					10	WS	Polyporoid fungi	HMJAU60803	
*Polyporus submelanopus* H.J. Xue & L.W. Zhou	*Polyporaceae*	*Polyporus*		√					1	WS	Polyporoid fungi	HMJAU60804	
*Polyporus umbellatus* (Pers.) Fr.	*Polyporaceae*	*Polyporus*	√						4	SS	Polyporoid fungi	HMJAU60805	
*Psathyrella delineata* (Peck) A.H. Sm.	*Psathyrellaceae*	*Psathyrella*				√			1	WS	Agarics	HMJAU60806	
*Psathyrella typhae* (Kalchbr.) A. Pearson & Dennis	*Psathyrellaceae*	*Psathyrella*					√		4	WS	Agarics	HMJAU60807	
*Pseudolaccaria fellea* (Peck) Vizzini, Matheny & Consiglio & M. Marchetti	*Callistosporiaceae*	*Pseudolaccaria*			√				3	WS	Agarics	HMJAU60808	
*Pseudoplectania nigrella* (Pers.) Fuckel	*Sarcosomataceae*	*Pseudoplectania*			√				5	SS	Larger Ascomytetes	HMJAU60809	
*Pulveroboletus macrosporus* G. Wu & Zhu L. Yang	*Boletaceae*	*Pulveroboletus*				√			1	EM	Boletes	HMJAU60810	
*Ramaria cokeri* R.H. Petersen	*Gomphaceae*	*Ramaria*		√		√			2	EM	Coral fungi	HMJAU60811	
*Ramaria fennica* (P. Karst.) Ricken	*Gomphaceae*	*Ramaria*						√	1	EM	Coral fungi	HMJAU60812	
*Ramaria marrii* Scates	*Gomphaceae*	*Ramaria*	√						2	EM	Coral fungi	HMJAU60813	
*Ramaria sanguinipes* R.H. Petersen & M. Zang	*Gomphaceae*	*Ramaria*		√					2	EM	Coral fungi	HMJAU60814	
*Ramaria stricta* (Pers.) Quél.	*Gomphaceae*	*Ramaria*		√					3	EM	Coral fungi	HMJAU60815	
*Rhodocollybia butyracea* (Bull.) Lennox	*Omphalotaceae*	*Rhodocollybia*	√		√				12	SS	Agarics	HMJAU60816	
*Russula aeruginea* Lindblad ex Fr.	*Russulaceae*	*Russula*				√			3	EM	Agarics	HMJAU60817	
*Russula amoena* Quél.	*Russulaceae*	*Russula*	√	√					2	EM	Agarics	HMJAU60818	
*Russula cyanoxantha* (Schaeff.) Fr.	*Russulaceae*	*Russula*		√					1	EM	Agarics	HMJAU60819	
*Russula emetica* (Schaeff.) Pers.	*Russulaceae*	*Russula*	√	√		√			18	EM	Agarics	HMJAU60820	
*Russula foetens* Pers.	*Russulaceae*	*Russula*		√		√			3	EM	Agarics	HMJAU60821	
*Russula furcata* Pers.	*Russulaceae*	*Russula*				√			3	EM	Agarics	HMJAU60822	
*Russula grata* Britzelm.	*Russulaceae*	*Russula*	√						2	EM	Agarics	HMJAU60823	
*Russula lakhanpalii* A. Ghosh, K. Das & R.P. Bhatt	*Russulaceae*	*Russula*				√			2	EM	Agarics	HMJAU60824	OL891479
*Russula paludosa* Britzelm.	*Russulaceae*	*Russula*	√			√			6	EM	Agarics	HMJAU60825	
*Russula risigallina* (Batsch) Sacc.	*Russulaceae*	*Russula*						√	3	EM	Agarics	HMJAU60826	
*Russula rosea* Pers.	*Russulaceae*	*Russula*	√	√					4	EM	Agarics	HMJAU60827	
*Russula senecis* S. Imai	*Russulaceae*	*Russula*				√			6	EM	Agarics	HMJAU60828	
*Russula sororia* (Fr.) Romell	*Russulaceae*	*Russula*				√			3	EM	Agarics	HMJAU60829	
*Russula veternosa* Fr.	*Russulaceae*	*Russula*	√						2	EM	Agarics	HMJAU60830	
*Russula vinosa* Lindblad	*Russulaceae*	*Russula*		√					2	EM	Agarics	HMJAU60831	
*Russula virescens* (Schaeff.) Fr.	*Russulaceae*	*Russula*	√						1	EM	Agarics	HMJAU60832	
Sarcodontia spumea (Sowerby) Spirin	*Meruliaceae*	*Sarcodontia*		√					3	WS	Polyporoid fungi	HMJAU60833	
*Schizophyllum commune* Fr.	*Schizophyllaceae*	*Schizophyllum*	√						4	WS	Agarics	HMJAU60834	
*Scleroderma areolatum* Ehrenb.	*Sclerodermataceae*	*Scleroderma*				√			4	EM	Gasteroid fungi	HMJAU60835	
*Sowerbyella rhenana* (Fuckel) J. Moravec	*Pyronemataceae*	*Sowerbyella*				√			5	EM	Larger Ascomytetes	HMJAU60836	
*Spathularia flavida* Pers.	*Cudoniaceae*	*Spathularia*			√				4	SS	Larger Ascomytetes	HMJAU60837	
*Stropharia rugosoannulata* Farl. ex Murrill	*Tubariaceae*	*Stropharia*		√					3	SS	Agarics	HMJAU60838	
*Suillus americanus* (Peck) Snell	*Suillaceae*	*Suillus*			√				1	EM	Boletes	HMJAU60839	
*Suillus granulatus* (L.) Roussel	*Suillaceae*	*Suillus*						√	1	EM	Boletes	HMJAU60840	
*Suillus grevillei* (Klotzsch) Singer	*Suillaceae*	*Suillus*			√		√		10	EM	Boletes	HMJAU60841	OL891494
*Suillus luteus* (L.) Roussel	*Suillaceae*	*Suillus*			√	√			4	EM	Boletes	HMJAU60842	OL891496
*Suillus placidus* (Bonord.) Singer	*Suillaceae*	*Suillus*			√				3	EM	Boletes	HMJAU60843	OL884444
*Suillus subaureus* (Peck) Snell	*Suillaceae*	*Suillus*	√						5	EM	Boletes	HMJAU60844	OL891495
*Suillus tomentosus* Singer, Snell & E.A. Dick	*Suillaceae*	*Suillus*	√						3	EM	Boletes	HMJAU60845	
*Sutorius brunneissimus* (W.F. Chiu) G. Wu & Zhu L. Yang	*Boletaceae*	*Sutorius*		√					1	EM	Boletes	HMJAU60846	
*Tengioboletus glutinosus* G. Wu & Zhu L. Yang	*Boletaceae*	*Tengioboletus*		√		√			5	EM	Boletes	HMJAU60847	
*Tremella Fuciformis* Berk.	*Tremellaceae*	*Tremella*	√						3	WS	Jelly fungi	HMJAU60848	
*Trichoderma rhododendri* (Jaklitsch) Jaklitsch & Voglmayr	*Hypocreaceae*	*Trichoderma*			√				3	WS	Larger Ascomytetes	HMJAU60849	
*Tricholoma matsutake* (S. Ito & S. Imai) Singer	*Tricholomataceae*	*Tricholoma*					√		4	EM	Agarics	HMJAU60850	
*Tricholoma psammopus* (Kalchbr.) Quél.	*Tricholomataceae*	*Tricholoma*		√	√				5	EM	Agarics	HMJAU60851	OL891480
*Tricholoma saponaceum* (Fr.) P. Kumm.	*Tricholomataceae*	*Tricholoma*			√				2	EM	Agarics	HMJAU60852	
*Tricholoma sejunctum* (Sowerby) Quél.	*Sarcoscyphaceae*	*Tricholoma*		√					4	EM	Agarics	HMJAU60853	OL891481
*Tricholoma stans* (Fr.) Sacc.	*Tricholomataceae*	*Tricholoma*				√			2	EM	Agarics	HMJAU60854	
*Tricholoma subacutum* Peck	*Tricholomataceae*	*Tricholoma*	√			√			2	EM	Agarics	HMJAU60855	
*Tricholoma ustale* (Fr.) P. Kumm.	*Tricholomataceae*	*Tricholoma*				√			1	EM	Agarics	HMJAU60856	OL891482
*Tricholoma ustaloides* Romagn.	*Tricholomataceae*	*Tricholoma*	√						1	EM	Agarics	HMJAU60857	
*Tricholomopsis rutilans* (Schaeff.) Singer	*Tricholomataceae*	*Tricholomopsis*						√	2	SS	Agarics	HMJAU60858	
*Tylopilus eximius* (Peck) Singer	*Boletaceae*	*Tylopilus*				√			1	EM	Boletes	HMJAU60859	OL891491
*Tylopilus felleus* (Bull.) P. Karst.	*Boletaceae*	*Tylopilus*					√		1	EM	Boletes	HMJAU60860	
*Tylopilus virens* (W.F. Chiu) Hongo	*Boletaceae*	*Tylopilus*	√						2	EM	Boletes	HMJAU60861	OL891493
*Tyromyces lacteus* (Fr.) Murrill	*Incrustoporiaceae*	*Tyromyces*	√						1	WS	Polyporoid fungi	HMJAU60862	
*Vanrija pseudolonga* (M. Takash., Sugita, Shinoda & Nakase) Weiß	*Cryptococcaceae*	*Vanrija*		√					1	EM	Boletes	HMJAU60863	
*Volvopluteus michiganensis* (A.H. Sm.) Justo & Minnis	*Pluteaceae*	*Volvopluteus*	√						1	SS	Boletes	HMJAU60864	
*Wynnea gigantea* Berk. & M.A. Curtis	*Wynneaceae*	*Wynnea*	√	√					5	SS	Larger Ascomytetes	HMJAU60865	
*Xerocomus ferrugineus* (Schaeff.) Alessio	*Boletaceae*	*Xerocomus*					√		1	EM	Boletes	HMJAU60866	
*Xerocomus magniporus* M. Zang & R.H. Petersen	*Boletaceae*	*Xerocomus*	√						1	EM	Boletes	HMJAU60867	
*Xeromphalina campanella* (Batsch) Kühner & Maire	*Mycenaceae*	*Xeromphalina*						√	1	WS	Agarics	HMJAU60868	
*Xylaria hypoxylon* (L.) Grev.	*Xylariaceae*	*Xylaria*		√					4	WS	Larger Ascomytetes	HMJAU60869	
*Xylaria polymorpha* (Pers.) Grev.	*Xylariaceae*	*Xylaria*	√						1	WS	Larger Ascomytetes	HMJAU60870	
	Note: Abbreviations: A = *Q. mongolica* forest; B = Broad-leaved forest; C = *P. koraiensis* and *Q. mongolica* mixed forest, with *P. koraiensis* as the dominant species; D = *P. koraiensis* and *Q. mongolica* mixed forest, with *Q. mongolica* as the dominant species; E = *P. koraiensis* forest; F = random collection; N = Number of fruiting bodies; ML = Mode of Life; SN = Specimen Number; EM = ectomycorrhizal; SS = soil saprotroph; WS = wood saprotroph; LS = litter saprotroph; DS = dung saprotroph; EI = endophyte-insect pathogen.												

## Appendix C. Species Scientific Names and Corresponding Abbreviations

**Species**	**Acronyms**	**Species**	**Acronyms**	**Species**	**Acronyms**	**Species**	**Acronyms**	**Species**	**Acronyms**
*Amanita altipes*	Ama1	*Cortinarius armillatus*	Cor2 (A)	*Inocybe assimilata*	Ino1 (A)	*Leccinum scabrum*	Lec2 (D)	*Russula vinosa*	Ru14
*Amanita chepangiana*	Ama2	*Cortinarius balaustinus*	Cor3 (A)	*Inocybe asterospora*	Ino2 (A)	*Leccinum versipelle*	Lec3 (A)	*Russula virescens*	Ru15 (A)
*Amanita excelsa*	Ama3 (D)	*Cortinarius bivelus*	Cor4	*Inocybe suaveolens*	Ino3	*Leucocybe connata*	Leu1 (A)	*Scleroderma areolatum*	Scl1
*Amanita flavipes*	Ama4	*Cortinarius caperatus*	Cor5 (A)	*Laccaria amethystina*	Lac1 (D)	*Lyophyllum decastes*	Lyo1 (A)	*Sowerbyella rhenana*	Sow1 (D)
*Amanita fuliginea*	Ama5	*Cortinarius cotoneus*	Cor6	*Laccaria laccata*	Lac2	*Lyophyllum infumatum*	Lyo2	*Suillus americanus*	Sui1
*Amanita hemibapha*	Ama6	*Cortinarius ectypus*	Cor7 (A)	*Lactarius albidocinereus*	Lact1 (D)	*Paxillus involutus*	Pax1	*Suillus grevillei*	Sui2
*Amanita ibotengutake*	Ama7	*Cortinarius flammeouraceus*	Cor8 (A)	*Lactarius brunneoviolascens*	Lact2 (A)	*Paxillus orientalis*	Pax2 (A)	*Suillus luteus*	Sui3
*Amanita oberwinklerana*	Ama8	*Cortinarius hesleri*	Cor9 (A)	*Lactarius conglutinatus*	Lact3 (A)	*Phylloporus yunnanensis*	Phy1 (A)	*Suillus placidus*	Sui4
*Amanita orsonii*	Ama9	*Cortinarius pholideus*	Cor10 (D)	*Lactarius deterrimus*	Lact4	*Pulveroboletus macrosporus*	Pul1 (D)	*Suillus subaureus*	Sui5 (A)
*Amanita pallidocarnea*	Ama10	*Cortinarius sanguineus*	Cor11 (D)	*Lactarius flavidus*	Lact5 (A)	*Ramaria cokeri*	Ram 1	*Suillus tomentosus*	Sui6 (A)
*Amanita pallidorosea*	Ama11 (A)	*Cortinarius subbalaustinus*	Cor12 (A)	*Lactarius glyciosmus*	Lact6 (A)	*Ramaria marrii*	Ram2 (A)	*Sutorius brunneissimus*	Sut1
*Amanita rimosa*	Ama12 (A)	*Cortinarius torvus*	Cor13 (A)	*Lactarius hirtipes*	Lact7 (A)	*Ramaria sanguinipes*	Ram 3	*Tengioboletus glutinosus*	Ten1
*Amanita rubescens*	Ama13	*Craterellus cornucopioides*	Cra1(A)	*Lactarius lilacinus*	Lact8 (A)	*Ramaria stricta*	Ram 4	*Tricholoma matsutake*	Tri1
*Amanita subglobosa*	Ama14 (A)	*Gomphidius maculatus*	Gom1	*Lactarius pallidus*	Lact9	*Russula aeruginea*	Rus1 (D)	*Tricholoma psammopus*	Tri2
*Amanita vaginata*	Ama15	*Harrya chromapes*	Har1 (A)	*Lactarius piperatus*	Lact10 (A)	*Russula amoena*	Rus2	*Tricholoma saponaceum*	Tri3
*Amanita virosa*	Ama16	*Hebeloma birrus*	Heb1 (A)	*Lactarius proximellus*	Lact11	*Russula cyanoxantha*	Rus3	*Tricholoma sejunctum*	Tri4
*Boletus aereus*	Bol1	*Helvella crispa*	Hel1	*Lactarius pubescens*	Lact12 (A)	*Russula emetica*	Rus4	*Tricholoma stans*	Tri5 (D)
*Boletus edulis*	Bol2	*Helvella elastica*	Hel2	*Lactarius subvellereus*	Lact13 (A)	*Russula foetens*	Rus5	*Tricholoma subacutum*	Tri6
*Bothia castanella*	Bot1 (A)	*Helvella macropus*	Hel3	*Lactarius torminosus*	Lact14 (D)	*Russula furcata*	Rus6 (D)	*Tricholoma ustale*	Tri7 (D)
*Butyriboletus regius*	But1 (A)	*Humaria hemisphaerica*	Hum1	*Lactarius trivialis*	Lact15 (A)	*Russula grata*	Rus7(A)	*Tricholoma ustaloides*	Tri8 (A)
*Butyriboletus roseoflavu*	But2	*Hydnellum aurantiacum*	Hyd1	*Lactarius vietus*	Lact16 (A)	*Russula lakhanpalii*	Rus8 (D)	*Tylopilus eximius*	Tyl1 (D)
*Chalciporus piperatus*	Cha1	*Hydnellum peckii*	Hyd2	*Lactarius volemus*	Lact17 (A)	*Russula paludosa*	Rus9	*Tylopilus felleus*	Tyl2
*Chroogomphus helveticus*	Chr1	*Hygrophorus conicus*	Hyg1	*Lactarius zonarius*	Lact18	*Russula rosea*	Rus10	*Tylopilus virens*	Tyl3 (A)
*Coltricia crassa*	Col1	*Hygrophorus nemoreus*	Hyg2	*Lactifluus bertillonii*	Lacti1 (A)	*Russula senecis*	Rus11 (D)	*Vanrija pseudolonga*	Van1
*Coltricia strigosipes*	Col2 (A)	*Hygrophorus pudorinus*	Hyg3 (A)	*Lactifluus pilosus*	Lacti2 (A)	*Russula sororia*	Rus12 (D)	*Xerocomus ferrugineus*	Xer1
*Cortinarius anomalus*	Cor1 (D)	*Hygrophorus russula*	Hyg4 (A)	*Leccinum aurantiacum*	Lec1 (A)	*Russula veternosa*	Rus13 (A)	*Xerocomus magniporus*	Xer2 (A)
	Note: Abbreviations: Overlapping species labels are marked and noted in parentheses. A = *Q. mongolica* forest; D = *P. koraiensis* and *Q. mongolica* mixed forest, with *Q. mongolica* as the dominant species.								
